# Betaine‐Conjugated ß‐Peptide Foldamers: Influence of Quaternary Charge on Self‐Organization and MorphologyFormation

**DOI:** 10.1002/open.202500340

**Published:** 2025-09-26

**Authors:** Nikolett Varró, Eszter Erdei, Dóra Bogdán, Eszter Kalydi, Ruth Deme, Balázs Balogh, Imola Cs. Szigyártó, Tamás Beke‐Somfai, Zoltán Varga, Pál Szabó, István M. Mándity

**Affiliations:** ^1^ Department of Organic Chemistry Semmelweis University Hőgyes Endre utca 7 1092 Budapest Hungary; ^2^ Artificial Transporters Research Group Institute of Materials and Environmental Chemistry HUN‐REN Research Centre for Natural Sciences Magyar tudósok, körútja 2 1117 Budapest Hungary; ^3^ Biomolecular Self‐assembly Research Group Institute of Materials and Environmental Chemistry HUN‐REN Research Centre for Natural Sciences Magyar tudósok körútja 2. H‐1117 Budapest Hungary; ^4^ Biological Nanochemistry Research Group Institute of Materials and Environmental Chemistry HUN‐REN Research Centre for Natural Sciences Magyar tudósok körútja 2 H‐1117 Budapest Hungary; ^5^ Department of Physical Chemistry and Materials Science Faculty of Chemical Technology and Biotechnology Budapest University of Technology and Economics Műegyetem rkp., 3. H‐1111 Budapest Hungary; ^6^ MS Metabolomics Laboratory Cetre for Structural Study HUN‐REN Research Centre for Natural Sciences Magyar tudósok körútja 2 H‐1117 Budapest Hungary

**Keywords:** ß‐amino acid, betaine, folding, peptide, quaternary charge

## Abstract

The modification of well‐known *β*‐peptide helices has been achieved by the application of N‐terminal betaine conjugation. The 3D self‐organization of oligomers formed by [1*S*,2*S*]‐2‐aminocyclopentanecarboxylic acid (ACPC), [1*R*,2*R*]‐2‐aminocyclohexanecarboxylic acid (ACHC), and an alternating heterochiral homooligomer of [1*S*,2*S*]‐ACPC and [1*R*,2*R*]‐ACPC was studied. Results of NMR, ECD, FT‐IR, and molecular modeling showed that for [1*S*,2*S*]‐ACPC pentamer (**1**), the betaine conjugation did not affect the folding to an H12 helix. In contrast, for the [1*R*,2*R*]‐ACHC tetramer (**2**) betaine conjugation notably influenced the folding, and an H14 helix was observed instead of the expected H10 helix. In addition, this is the first observation of self‐association for an H12 helix forming *β*‐peptide. Based on TEM images, this association leads to vesicle morphologies. For the alternating heterochiral homooligomer [1*S*,2*S*]‐ACPC and the [1*R*,2*R*]‐ACPC pentamer (**3**), betaine conjugation enhances the solubility of the system. Moreover, the formation of an expected E‐strand can be anticipated, since self‐association was found in the form of a fibrin net‐like structure in TEM images. Betaine conjugates described herein open a new area of bioactive peptide foldamer construction, since the introduced quaternary charges may lead to important receptor‐ligand interactions, while potential material science applications can also be realized.

## Graphical abstract

N‐terminal betaine conjugation modifies *β*‐peptide helices, influencing folding and self‐organization. For [1S, 2S]‐ACPC, folding to an H12 helix was unaffected, while [1R, 2R]‐ACHC formed an H14 helix instead of H10. Alternating heterochiral oligomers made of enantiomeric pairs of trans‐ACPC showed enhanced solubility and E‐strand formation. These conjugates enable bioactive foldamer construction with potential biomedical and material science applications through quaternary charge introduction.

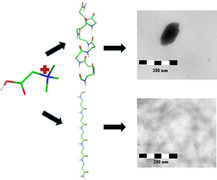

## 1 Introduction

In the realm of scientific inquiry, there exists a fascinating array of non‐natural compounds with peptide‐like characteristics, showcasing an impressive spectrum of structural variability and wide‐ranging utility across diverse scientific disciplines.^[^ ^1^ ^]^ Notably, *β*‐peptides, characterized by their composition of *β*‐amino acids, stand out as a particularly scrutinized subset within this landscape, representing artificial constructs renowned for their inherent self‐organizing properties. Classified as foldamers,^[^ ^2^ ^]^ these molecular entities possess an intriguing propensity to adopt stable 3D conformations even when consisting of relatively short sequences. Their versatility extends to the formation of various secondary structures, encompassing helices,^[^ ^1d^ ^,^ ^1f^ ^,^ ^3^ ^]^ strands,^[^ ^4^ ^]^ and intricate hairpins,^[^ ^4b^ ^,^ ^4c^ ^,^ ^5^ ^]^ while also exhibiting a remarkable capacity for the assembly of higher‐order tertiary architectures such as vesicles^[^ ^1a^ ^,^ ^1e^ ^,^ ^3a^ ^,^ ^4d^ ^,^ ^6^ ^]^ and nanofibers.^[^ ^4d^ ^,^ ^7^ ^]^

In terms of interdisciplinary research, foldamers have emerged as focal points of considerable interest, captivating attention across a myriad of industrial sectors. Their versatile applications span various domains, ranging from biomedicine,^[^ ^1d^ ^,^ ^1e^ ^,^ ^6f^ ^,^ ^8^ ^]^ where they exhibit promise in modulating protein–protein interactions,^[^ ^9^ ^]^ to the forefront of nanotechnology,^[^ ^7c^ ^,^ ^7d^ ^]^ where their manipulation drives advancements in material chemistry. Furthermore, their utility extends to surface recognition^[^ ^8d^ ^,^ ^9b^ ^,^ ^10^ ^]^ within artificial self‐assemblies, unlocking new avenues for exploration.

Delving deeper into their potential, foldamers unveil an intricate tapestry of functionalities that transcend traditional boundaries. These molecular architectures present themselves as catalysts^[^ ^8b^ ^,^ ^11^ ^]^ and sensors,^[^ ^12^ ^]^ poised to revolutionize chemical processes and analytical methodologies.

These examples emphasize the importance of the folding of *β*‐peptides and self‐assemblies thereof to fulfill material chemical and biomedical functions beyond those of natural oligomers. The folding of *β*‐peptides has been fine‐tuned by various technologies, like backbone stereochemistry^[^ ^4a^ ^,^ ^4e^ ^,^ ^5a^ ^,^ ^13^ ^]^ and side‐chain shape.^[^ ^4b–d^ ^,^ ^6g^ ^,^ ^6h^ ^,^ ^14^ ^]^

In some cases, betaine, a zwitterionic molecule, has been utilized to modulate properties of peptides and proteins, including stability, solubility, and bioactivity. Incorporating betaine moieties into peptide sequences can confer enhanced resistance to proteolytic degradation, whereas betaine‐containing peptides exhibit enhanced aqueous solubility.^[^ ^15^ ^]^

Herein, we show that by the introduction of quaternary cationic charges to foldameric structures, fine‐tuning of solubility, folding, and self‐assembly is further feasible.

## 2 Results and Discussion

**Scheme 1:**
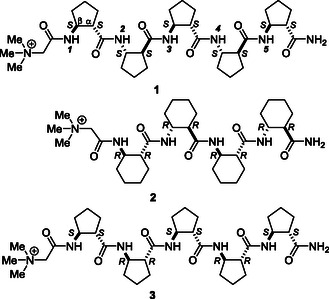
The chemical structures of betaine‐conjugated foldamers

The two archetypes of *β*‐peptide helices, the right‐handed H12 helix formed by the [1*S*,2*S*]‐2‐aminocyclopentanecarboxylic acid (ACPC) homochiral homooligomers^[^ ^16^ ^]^ and right‐handed H10 helix formed by the [1*R*,2*R*]‐2‐aminocyclohexanecarboxylic acid (ACHC) homochiral tetramer^[^ ^3a^ ^]^‐, are well characterized 3D constructs. Regarding the strand like structures, the alternating heterochiral homooligomers of [1*S*,2*S*]‐ACPC and [1*R*,2*R*]‐ACPC^[^ ^4e^ ^]^ previously published are a good example. In this study, we equipped the abovementioned oligomers with N‐terminal betaine; thus, betaine‐conjugated homochiral pentamer of [1*S*,2*S*]‐ACPC (**1**),betaine‐conjugated [1*R*,2*R*]‐ACHC tetramer (**2**), and betaine‐conjugated alternating heterochiral homooligomers of [1*S*,2*S*]‐ACPC and [1*R*,2*R*]‐ACPC (**3**) were created. The structures of the investigated betainiated compounds are shown in **Scheme** **1**.

The synthesis of oligomers **1** **–3** was carried out first under regular solid‐phase peptide synthesis (SPPS) conditions. However, due to the bulky nature of betaine possessing the quaternary positive charge, the desired products were not isolated. To overcome this problem, continuous‐flow solid‐phase peptide synthesis (CF‐SPPS) was utilized.^[^ ^17^ ^]^ This technique allows the use of low amino acid excesses, while complete conversion can be gained. It was already tested for the synthesis of *β*‐peptides,^[^ ^13c^ ^]^ however not for the coupling of bulky betaine on sterically hindered *β*‐amino acids. Under optimized conditions, compounds **1**–**3** were isolated in >96% crude purity and >91% yields.

**Figure 1:**
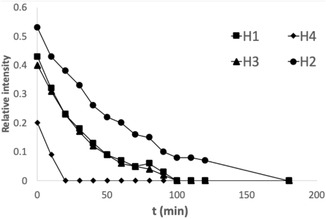
Time dependence of the NH/ND exchange for 4 mM solutions of **1** in CD_3_OD.

First, the 3D organization of oligomers **1**–**3** were assessed by the NH/ND exchange measured by a series of 1H NMR spectra in CD_3_OD in 4 mM concentration at 297 K. For oligomer **1** the time dependence of the residual NH signal intensities point to the fact that the corresponding atoms being considerably shielded from the solvent, due to the H‐bonding interactions (**Figure** **1**).

Proton resonances belonging to the C‐terminal amide disappeared immediately after dissolution, while other signals remained for a longer period of time. The exchange pattern observed is in good accordance with the structure.^[^ ^6g^ ^]^ The slowest exchange pattern was observed for NH^2^. It is in the center of the helix, which shields considerably the proton from the solvent. NH^2^ is followed by NH^1^ and NH^3^. The result of NH^3^ is not surprising, since a proton is still in the middle of the structure. The slow exchange of NH^1^, in turn, is somehow unexpected. However, it can be explained by the shielding effect of the bulky betaine part close to NH^1^. A considerably faster exchange was observed for NH^4^, while NH^5^ disappeared immediately after dissolution. This exchange pattern predicts the formation of an H12 helix, nonetheless, it is influenced by the self‐association of the helices and by the bulky nature of the N‐terminal betaine moiety.

For **2**, considerably slower exchange was observed. Namely, within 2 weeks, only 5–10% exchange was detected, and complete exchange was not reached even within 2 months. This result does not support the formation of an H10 helix; rather, it suggests the presence of an H14 fold and indicates potential self‐aggregation.^[^ ^3a^ ^]^

Considering **3**, the protic amide protons disappeared immediately after dissolution, suggesting the formation of an extended, strand‐like conformation.^[^ ^4e^ ^]^

**Figure 2:**
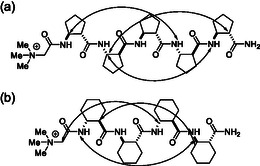
Long‐range NOE interactions for 1 a) in DMSO‐d6, and for 2 b) found in DMSO‐d6, CD3OH or water (90% H2O + 10% D2O).

To gain high‐resolution structural data, various NMR measurements, including COSY, TOCSY, and ROESY, in 4 mM CD_3_OD, DMSO‐*d*_*6*_, and water (90% H_2_O + 10% D_2_O) solutions were carried out at 297K. Full signal assignment for backbone protons was carried out for **1** and **2**, and long‐range NOEs specific for helical *β*‐peptide secondary structures were detected. The long‐range NOEs are shown in **Figure** **2**

For **1**, long‐range NOEs were found typical for H12 helices^[^ ^4c^ ^]^ in 4 mM concentration in CD_3_OD, DMSO‐*d*_*6*_, and water (90% H_2_O + 10% D_2_O) such as H_*N*_(4)–H_*β*_(1), H_*N*_(5)–H_*β*_(2), and H_*α*_(5)–H_*β*_(2). These NOE crosspeaks are unambiguous signs for the formation of H12 helix. Importantly, the structure tends to be stable in methanol, water, and even in chaotropic DMSO too at 4 mM concentration. The results suggest that the previously known H12 helix made by the homochiral pentamer of [1*S*,2*S*]‐ACPC is maintained, that is, betaine conjugation does not influence the helix‐forming property of H12.

For **2**, a different set of long‐range NOEs was observed in a 4 mM concentration in CD_3_OD, DMSO‐*d*_*6*_, and water (90% H_2_O + 10% D_2_O): H_*N*_(1)–H_*β*_(4); H_*α*_(1)–H_*β*_(4), and CH_2_(betaine) –H_*β*_(3). These long‐range interactions were found in 4 mM concentration for the solvents as follows: CD_3_OD, DMSO‐*d*_*6*_, and water (90% H_2_O + 10% D_2_O). This NOE crosspeak pattern is typical to the H14 helix.^[^ ^3a^ ^,^ ^4c^ ^]^ These results suggest that in this case the betaine influences the prevailing secondary structure. Namely, the H14 helix is formed rather than the H10 helix published previously.

However, for **3,** the signal dispersion was considerably low; thus, the backbone signal assignment could not be carried out, and it ruled out to look for long‐range NOEs to detect a prevailing secondary structure. The low signal dispersion is a considerable sign for strand‐like secondary structure formation.^[^ ^4a^ ^,^ ^4e^ ^]^ Importantly, due to the introduction of the charged quaternary ammonium ion group of the betaine moiety, compound **3** was soluble in CD_3_OD, DMSO‐*d*_*6*_, and water (90% H_2_O + 10% D_2_O) in a 4 mM concentration too. This opened the way for the utilization of further structure characterization techniques.

**Figure 3:**
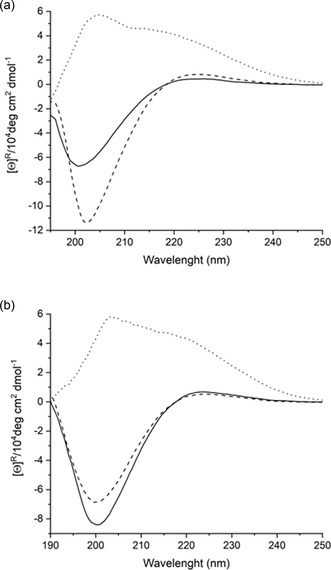
ECD curves of 1 mM solutions of **1** (solid), **2** (dashed) and **3** (dotted) in methanol a) and in water b). Intensities were normalized for the number of chromophores.

To further support the structure characterization data, electronic circular dichroism (ECD) measurements were carried out in methanol and water at a concentration of 1 mM at room temperature (**Figure** **3**).

The ECD curve of **1** shows a minor positive band at around 226 nm, while a considerable negative band can be observed at 200 nm in methanol solution. These data are typical for a right‐handed helical structure. Considering the NMR data, the most probable secondary is the H12 helix.^[^ ^4c^ ^]^ In aqueous solution, the same low‐intensity positive band is maintained at 223 nm. However, the intensity of the negative band considerably increased at 201 nm compared to those of methanol solutions. These data suggest that the same helical is maintained in water too. Surprisingly, the stability of the helix increased. This fact points to a potential solvent driven self‐association.

For compound **2,** there is positive band at 225 nm and a negative band of higher intensity at 202 nm in methanol. These slight bathochromic shift suggest the presence of a more compact helical fold; thus, the ECD curve indicates the formation of a compact helical conformation. Taking into account the NMR data, the H14 helix formation is most considered in methanol solution.^[^ ^4c^ ^]^ In water solution, the positive band of compound **2** appears at 224 nm, and the negative band moves to 200 nm. In this case, however, the intensity is considerably lower than those in the methanol solution. This pattern further supports the formation of the same helical structure than those for methanol solution, while it can be stated that the solvent influences the shape of the CD curve. The origin of these minor changes in the CD curve might indicate the potential self‐association of the formed helices.

The ECD curve measured for **3** shows a completely different pattern. Importantly, the curve is not crossing the *x* axis, which is a considerable sign for strand‐like structure formation.^[^ ^4c^ ^]^ The curve measured in methanol exhibits positive bands at 212 nm and at 204 nm. A similar pattern can be observed in water, while the intensity of the bands in both solutions tends to be the same. Thus, it can be concluded that the solvent does not have any drastic influence on the prevailing self‐organization of the strand‐like structure. The introduction of the quaternary charge, by the conjugation of betaine to *β*‐peptide, contributed to water and methanol soluble compound, where ECD curves could be gained from both solvents.

These conformational alterations were also detected by FT‐IR spectroscopy. The amide I band is originating mainly from carbonyl stretching vibrations of the peptide backbone.

**Figure 4:**
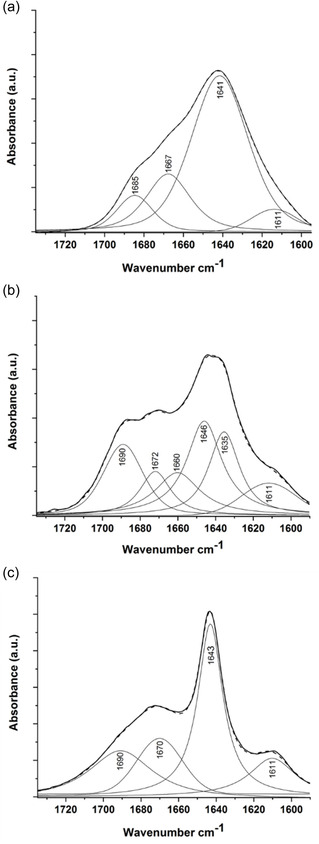
FT‐IR spectra in the amide I region of **1** a), **2** b), and **3** c). Spectra were recorded for dry film samples from 1 mM peptide solution and normalized by the area. The solid and dash lines denote the measured and the fitted curves, respectively, whereas thin curves correspond to individual band components (using second derivative IR spectra and Fourier self deconvolution).

The most intense band of the deconvoluted amide I of **1** (**Figure** **4**a) is ≈1641 cm^−1^ which could be assigned to strong intramolecular hydrogen bonds.^[^ ^3a^ ^,^ ^4c^ ^]^ This fact supports the helical self‐association of the peptide. The two shoulders ≈ 1685 cm^−1^ and 1667 cm^−1^ can be related to weaker hydrogen bonds, like the C‐terminal amide bond. According to our knowledge, no FT‐IR spectrum is available for the H12 helix; however, the gained results underline the formation of a stable helical conformation.

In case of **2**, the deconvoluted amide band show characteristics vibrations of C = O groups ≈1646, 1674, and 1690 cm^−1^ involved or not in H‐bonding, respectively. This finding supports the results gained by NMR measurements that the conjugation of the betaine unit shifts the folding of peptide to an H14 helix, instead of the expected H10 helices.

In contrast, for **3,** the spectral pattern shows somewhat a different behavior. The sharper band ≈1643 cm^−1^ with the presence of a shoulder ≈1690 cm^−1^ can also be assigned to stronger and weaker hydrogen bonds involved in peptide folding. The shoulder ≈1611 cm^−1^ suggests the presence of intermolecular hydrogen bonds between *β*‐sheets^[^ ^18^ ^]^ supporting the formation of strand‐like structure.

**Figure 5:**
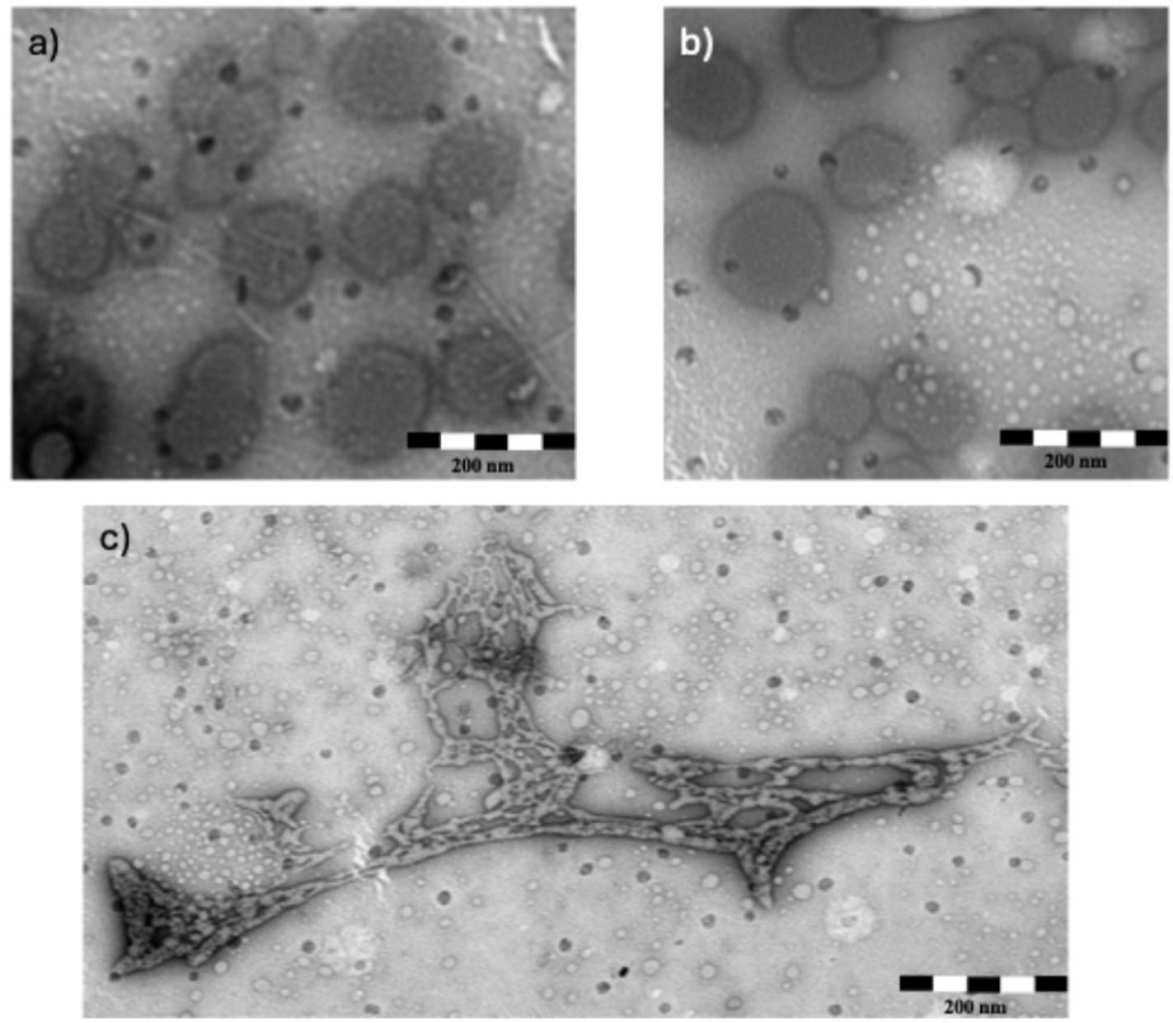
TEM images of vesicles observed after dissolution and sonication of 4 mM solutions of **1** a), **2** b), and **3** c) in water.

The extremely prolonged NH/ND exchange observed for **2** and the FT‐IR results obtained for **3** indicate the potential self‐association of the betaine‐conjugated *β*‐peptides. Consequently, transmission electron microscopy (TEM) was used to analyze the self‐association phenomena in water solution at 4 mM concentration. The images were recorded after dissolution and sonication (**Figure** **5**).

Surprisingly, for helix‐forming oligomers **1** and **2,** vesicle formation was observed even in water as solvent.^[^ ^3a^ ^,^ ^4c^ ^,^ ^4d^ ^,^ ^6g^ ^,^ ^6h^ ^]^ Importantly, this is the first observation of an H12 helix‐forming *β*‐peptide to show self‐association in the form of vesicles. The size of the vesicles is in the range of 130–200 nm as shown in Figure 5a.

Importantly, compound **1**, which adopts an H12 helical conformation, was found to self‐associate into vesicles with diameters ranging from 130 to 180 nm (Figure 5a). To the best of our knowledge, this represents the first observation of vesicle formation from H12 helical structures. In the case of compound **2**, vesicles with an average diameter similar than those of for **1** were observed (Figure 5b). Notably, there was no need for a time‐consuming incubation period, since vesicles were detected immediately after dissolution and sonication. This observation further supports the view that by the introduction of quaternary positive charges the vertical amphiphilicity of *β*‐peptides can be increased, resulting in higher tendency for self‐association manifesting in vesicle formation. These results suggest that by betaine conjugation not only the secondary structure, but the formation of tertiary structural element formation can be fine‐tuned. Importantly, the self‐association was found in water, which is a considerably more biorelevant solvent.

The TEM images recorded for **3** showed a significantly different pattern (Figure 5c). Vesicle formation was not observed, rather, nanosized fibrils were found. This result is in accordance with literature data. For example, peptides forming strand‐like secondary structure have strong propensity for nanosized fibril formation.^[^ ^4d^ ^,^ ^4e^ ^]^ Note, however, that this is the first observation for the formation of a *β*‐peptide fibril formation in aqueous medium. Moreover, the observed fibrils form a concatenated fibrinous net‐like structure.

**Figure 6:**
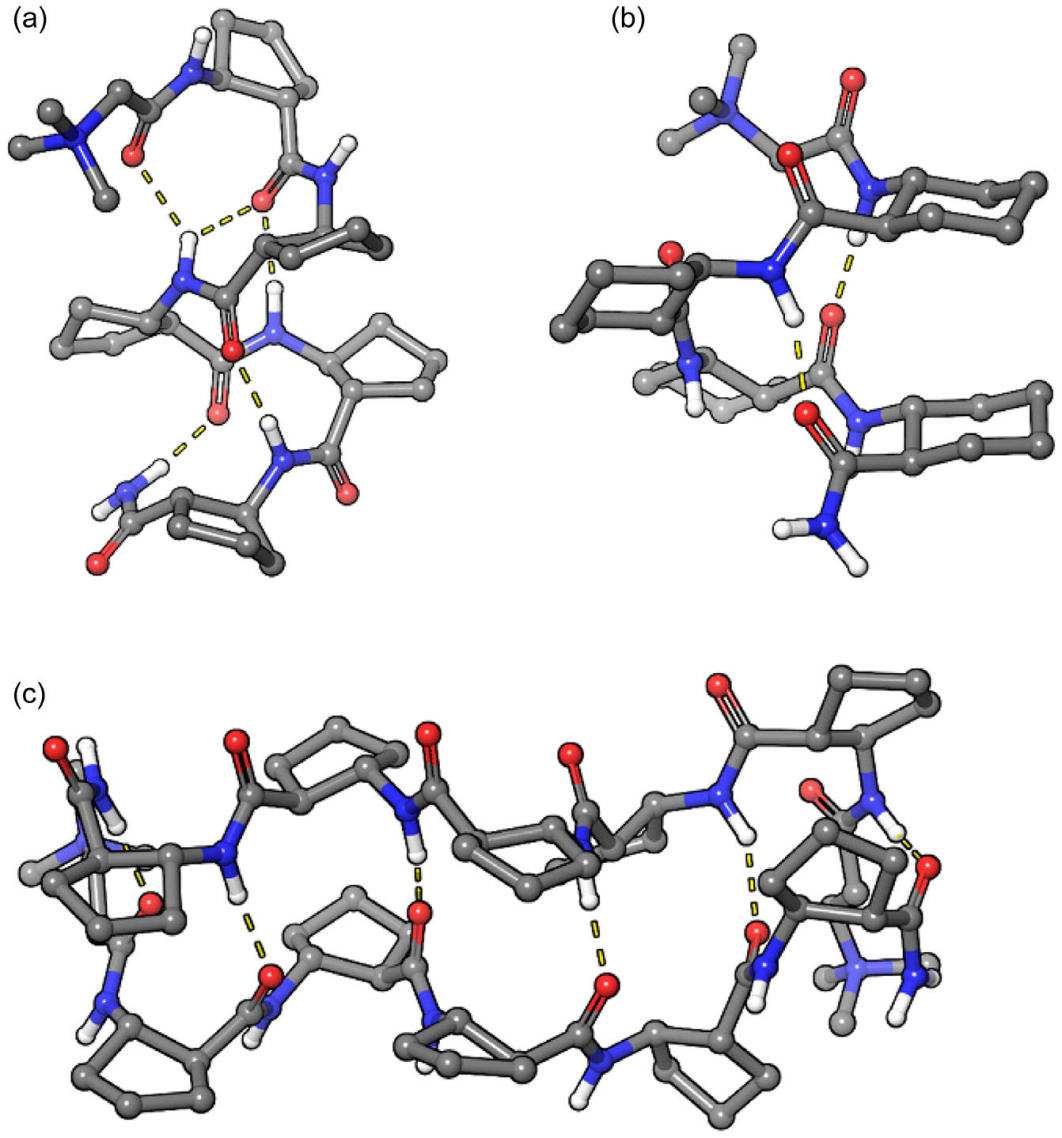
Optimized ab initio geometry of **1** a), **2** b), and **3** c) at the B3LYP/6‐311G** level of theory.

To further support the previously gained experimental results, theoretical calculations were carried out. Quantum mechanical DFT (density functional theory) calculations, namely geometry optimizations, were carried out at the B3LYP/6‐311G** level of theory with the Jaguar software package.^[^ ^19^ ^]^ The calculations suggest the formation of an H12 helix for **1** and an H14 helix for **2**. For **3**, the calculation supported the formation of an antiparallel *β*‐sheet formation by the self‐association of E‐strands. The optimized geometries are shown in **Figure** **6**.

It can be concluded that betaine conjugation significantly influenced the folding, self‐assembly, and solubility of a set of *β*‐peptide foldamers. The oligomers were synthetized on solid support by the use of a prominently economic continuous‐flow technology, which allowed the use of only 1.5‐fold of excess of amino acids.

In the case of the [1*S*,2*S*]‐ACPC pentamer (**1**), betaine conjugation did not influence the folding of an H12 helix. However, the self‐association of this oligomer was considerably facilitated. In water as solution, vesicles in the range of 130−180 nm were observed in TEM images.

For the [1*R*,2*R*]‐ACHC tetramer (**2**), betaine conjugation significantly influenced the folding. Note, however, that formation of the H14 helix was observed instead of the expected H10 helix. Betaine conjugation considerably increased the self‐association property, and immediately after dissolution and sonication, vesicles with a diameter of ca. 200 nm were observed in TEM images.

For the alternating heterochiral [1*S*,2*S*]‐ACPC and [1*R*,2*R*]‐ACPC pentamer (**3**), betaine conjugation increases the solubility of the system. The prevailing secondary structure is the expected E‐strand, which is capable of self‐association in the form of amyloid like fibrils. However, betaine conjugation modified slightly this behavior, and a concatenated, fibrin net‐like structure was observed.

For the investigated betaine‐conjugated oligomers, self‐association was found in aqueous medium. This fact opens the window for biomedical and material chemical utilization of *β*‐peptide foldamers containing alicyclic side‐chain.

## 3 Experimental Section

3.1

3.1.1

##### Peptide Synthesis

Peptide chains were extended on a Tentagel R RAM resin (0.20 mmol g^−1^). For CF experiments, a modular CF apparatus was assembled, consisting of a cylindrical PEEK column (with internal dimensions of 250 × 4 mm) filled with the resin‐loaded amino acid (350 mg), a semipreparative pump (JASCO PU‐4086), an HPLC autosampler (JASCO AS‐4150), a column oven (JASCO CO‐4060), two line‐selecting valve units (JASCO HV‐4380), and a backpressure regulator. A coupling mixture, consisting of 1.5 equivalents of Fmoc‐protected *β*‐amino acid and 1.5 equivalents of OxymaPure as coupling reagent dissolved in DMF and 1.5 equivalents of DIC, was mixed by the autosampler. The coupling mixture has been prepared immediately before the coupling reaction. Coupling reactions were carried out at the optimized reaction conditions, 85 bar pressure, 65 °C temperature, and 0.2 mL min^−1^ flow rate. For Fmoc deprotection, the solution of 2 mL of 2% DBU 2% piperidine in DMF has been used. Between two chemical steps DMF was used for washing for 5 min.

##### NMR Experiments

NMR measurements for signal assigment were carried out on a Bruker Avance III 500 MHz spectrometer equipped with a cryo probe head. Peptide samples (4 mM) were prepared in H_2_O/D_2_O 90:10 v/v, DMSO‐*d*_*6*_, or CD_3_OH and transferred into 5 mm NMR sample tubes. For the ROESY spinlock, a mixing time of 300 ms was used; the number of scans was 16, and roesyesgpph or roesyph.2 (DMSO‐*d*_*6*_) pulse sequence was applied. The TOCSY measurement was performed with the mlevesgpph or mlevph (DMSO‐*d*_*6*_) sequence, with a mixing time of 150 ms; the number of scans was 16. For all 2D spectra, 4k time domain points and 512 increments were applied. T2 relaxation experiments were carried out by cpmg_esgp2d or cpmg (DMSO‐*d*_*6*_) pulse sequence; the relaxation delays were incremented in the following order: 1, 2, 4, 8, 16, 32, 64, 128, 256, 512, and 1024 ms.

The NH/ND exchange was recorded on a Varian Mercury 400 spectrometer equipped with ATB PFG probe head. The samples were prepared in 4 mM concentration in CD_3_OD and transferred into standard 5 mm NMR sample tubes. The recording was started 10 min after complete solubilization. The ^1^H spectra were recorded using 64 scans with 45° pulse and 5 s relaxation delay.

##### CD Measurements

CD spectra were measured on a Jasco J‐1500 spectropolarimeter at 25°C in a 0.1 cm path length regtangular quartz cuvette (Hellma, Plainview, NY) in a continuous scanning mode between 190 and 250 nm at a rate of 50 nm min^−1^, with a data pitch of 0.5 nm, a response time of 4 s, a 1 nm bandwidth, and 3 times accumulation for each sample. The baseline spectrum recorded with the solvent was subtracted from the raw data. The concentration of the sample solutions in ultrapure water and PBS was 1 mM. Molar circular dichroism is given in mol^−1 ^cm^−1^.

##### ATR‐FTIR Measurement

A Varian 2000 FTIR Scimitar spectrometer (Varian Inc., Palo Alto, CA) was used for FTIR spectroscopic measurements. The spectrometer is fitted with a liquid nitrogen‐cooled mercury‐cadmium‐telluride (MCT) detector with a ‘Golden Gate’ single reflection diamond ATR accessory (Specac Ltd., Orpington, U.K.). On the diamond ATR surface, 3 µL of the sample was mounted, and the spectrum was accumulated (2 cm^−1^ resolution and 64 scans) for the dry film after gradual evaporation of the buffered solvent under ambient conditions. ATR correction for every data acquisition, buffer subtraction, and baseline corrections were performed. The GRAMS/32 software package (Galactic Inc.) was used for all spectral manipulations.

##### TEM Measurement

The peptides were dissolved in water to a concentration of 1 mM, and the solution was sonicated for 5 min. Drops of 5 µL of solutions were placed onto Formvar‐coated 200‐mesh copper grids (Ted Pella Inc, USA) and dried in the air at 25°C for 10 min. Specimens were studied with a MORGAGNI 268D transmission electron microscope (FEI, Eindhoven, The Netherlands) operated at 80 kV and equipped with a Quemesa 11‐megapixel bottom‐mounted CCD camera (Emsis GmbH, Germany).

##### Model Building and Theoretical Calculations

The structures were drawn with Schrödinger's Maestro 2D Sketcher, the hydrogen atoms were added, and the structures were quickly optimized into 3D geometry. A conformational search for each compound was completed with the MacroModel package ‘Energy minimization’ option using the OPLS4 force field with ‘no solvent’ option with the default settings. In the case of dimer **3**, in order to maintain the interaction between the two chains, ‘frozen’ atom constraints were applied on the corresponding hydrogen and oxygen atoms participating in the H‐bonds. The output structures of the minimization of each compound were used as initial geometries for further calculations.

The Jaguar geometry optimization calculations were completed in two steps. First, the Restricted Hartree–Fock (RHF) method was applied with 3‐21G basis set in a vacuum. Second, the density functional theory (DFT) method (B3LYP‐D3 functional) was applied with 6‐311G** basis set in vacuum. In the case of dimer **3**, implicit water was necessary in order to maintain chain‐like conformation, and the polarizable continuum model (PCM) solvation model was applied.

The software packages as follows were used: Maestro (v13.8): Release 2023‐4, Schrödinger, LLC, New York, NY, 2023; MacroModel (v14.2): Release 2023‐4, Schrödinger, LLC, New York, NY, 2023; and Jaguar (v12.2): Release 2023‐4, Schrödinger, LLC, New York, NY, 2023.

## Conflict of Interest

The authors declare no conflict of interest.

## Supporting information

Supplementary Material

## Data Availability

The data that support the findings of this study are available in the supplementary material of this article.
