# Supplementary material for: Betaine‐Conjugated ß‐Peptide Foldamers: Influence of Quaternary Charge on Self‐Organization and MorphologyFormation
Source: ChemistryOpen. 2025 Sep 26;14(12):e202500340. doi: 10.1002/open.202500340 (PMC12680565; doi:10.1002/open.202500340)
Supplement: Supplementary file 1 — Supplementary Material [file OPEN-14-e202500340-s001.pdf]

Betaine-conjugated β-peptide foldamers, influence of quaternary charge on self-organization and morphology formation

Nikolett Varró [a], Eszter Erdei [a,b], Dóra Bogdán [a,b], Eszter Kalydi [b], Ruth Deme [a,b], Balázs Balogh [b], Imola Cs. Szigyártó [c], Tamás Beke-Somfai [c], Zoltán Varga [d,e], Pál Szabó and István Mándity [a,b]*

Artificial Transporters Research Group, Institute of Materials and Environmental Chemistry, HUN-REN Research Centre for Natural Sciences, Magyar tudósok körútja 2., 1117 Budapest, Hungary

Department of Organic Chemistry, Semmelweis University, Hőgyes Endre utca 7., 1092 Budapest, Hungary

Biomolecular Self-assembly Research Group, HUN-REN Research Centre for Natural Sciences, Institute of Materials and Environmental Chemistry, Magyar tudósok körútja 2., H-1117 Budapest, Hungary

Biological Nanochemistry Research Group, HUN-REN Research Centre for Natural Sciences, Institute of Materials and Environmental Chemistry, Magyar tudósok körútja 2., H-1117 Budapest, Hungary

Department of Physical Chemistry and Materials Science, Faculty of Chemical Technology and Biotechnology, Budapest University of Technology and Economics, Műegyetem rkp. 3., H-1111 Budapest, Hungary

MS Metabolomics Laboratory, HUN-REN Research Centre for Natural Sciences, Cetre for Structural Study, Magyar tudósok körútja 2., H-1117 Budapest, Hungary

Supporting information

Table of content

Experimental methods 4

Peptide synthesis: 4

NMR experiments: 4

CD measurements: 5

ATR-FTIR measurement: 5

TEM measurement: 5

Model building and theoretical calculations 6

Peptide synthesis – HPLC-MS data 7

Figure S1. HPLC-MS for compound **1** 7

Figure S2. HPLC-MS for compound **2** 8

Figure S3. HPLC-MS for compound **3** 9

NMR results 10

Figure S4. Time dependence of the NH/ND exchange for 4 mM solutions of 1 in CD_3OD 10

Figure S5. Long-range NOE interactions for 1 (a) in DMSO-d_6, and for 2 (b) found in DMSO-d_6, CD_3OH or water (90% H_2O + 10% D_2O) 10
Figure S6. Assignment of backbone Hs of compound **1** in CD_3OH 11
Figure S7. ^{1}H NMR spectrum of compound **1** in CD_3OH 11
Figure S8. TOCSY NMR spectrum of compound **1** in CD_3OH 11
Figure S9. ROESY NMR spectrum of compound **1** in CD_3OH 12
Figure S10. Assignment of backbone Hs of compound **1** in H_2O/D_2O 9:1 12
Figure S11. ^{1}H NMR spectrum of compound **1** in H_2O/D_2O 9:1 12
Figure S12. TOCSY NMR spectrum of compound **1** in H_2O/D_2O 9:1 13
Figure S13. ROESY NMR spectrum of compound **1** in H_2O/D_2O 9:1 13
Figure S14. Assignment of backbone Hs of compound **1** in DMSO-d_6 13
Figure S15. ^{1}H NMR spectrum of compound **1** in DMSO-d_6 14
Figure S16. TOCSY NMR spectrum of compound **1** in DMSO-d_6 14
Figure S17. ROESY NMR spectrum of compound **1** in DMSO-d_6 15
Figure S18. Assignment of backbone Hs of compound **2** in CD_3OH 15
Figure S19. ^{1}H NMR spectrum of compound **2** in CD_3OH 15
Figure S20. TOCSY NMR spectrum of compound **2** in CD_3OH 16
Figure S21. ROESY NMR spectrum of compound **2** in CD_3OH 16
Figure S22. Assignment of backbone Hs of compound **2** in H_2O/D_2O 9:1 16
Figure S23. ^{1}H NMR spectrum of compound **2** in H_2O/D_2O 9:1 17
Figure S24. TOCSY NMR spectrum of compound **2** in H_2O/D_2O 9:1 17
Figure S25. ROESY NMR spectrum of compound **2** in H_2O/D_2O 9:1 18
Figure S26. Assignment of backbone Hs of compound **2** in DMSO-d_6 18
Figure S27. 1H NMR spectrum of compound **2** in DMSO-d_6 18
Figure S28. TOCSY NMR spectrum of compound **2** in DMSO-d_6 19
Figure S29. ROESY NMR spectrum of compound **2** in DMSO-d_6 19
Figure S30. ^{1}H NMR spectrum of compound **3** in CD_3OH 20
Figure S31. Time-dependent series of ^{1}H NMR spectra of compound **1**, showing the NH/ND exchange in CD_3OD at 297 K. The concentration of the sample was 4 mM. 21
TEM images 22
Figure S32. TEM image of vesicles observed after dissolution and sonication of 4 mM solutions of **1**. 22
Figure S33. TEM image of vesicles observed after dissolution and sonication of 4 mM solutions of **1**. 22
Figure S34. TEM image of vesicles observed after dissolution and sonication of 4 mM solutions of **1**. 23

Figure S35. TEM image of vesicles observed after dissolution and sonication of 4 mM solutions of **2**. 23

Figure S36. TEM image of vesicles observed after dissolution and sonication of 4 mM solutions of **2**. 24

Figure S37. TEM image of vesicles observed after dissolution and sonication of 4 mM solutions of **2**. 24

Figure S38. TEM image of vesicles observed after dissolution and 15 minute long sonication of 4 mM solutions of **2**........ 25

Figure S39. TEM image of vesicles observed after dissolution and sonication of 4 mM solutions of **3**. 25

Figure S40. TEM image of vesicles observed after dissolution and sonication of 4 mM solutions of **3**. 26

Figure S41. TEM image of vesicles observed after dissolution and sonication of 4 mM solutions of **3**. 27

HR-MS data........ 28

Figure S42. HR-MS spectrum of **1.** 28

Figure S43. HR-MS spectrum of **2.** 29

Figure S44. HR-MS spectrum of **3.** 30

Computational data at the B3LYP/6-311G** level of theory 31

Cartesian coordinates for **1** E(B3LYP/6-311G**) = -2203.92008782421 a.u 31

Cartesian coordinates for **2** E(B3LYP/6-311G**) = -1997.12887018855 a.u. 33

Cartesian coordinates for the dimer of **3** E(B3LYP/6-311G**) = -4486.79471300043 35

Experimental methods

Peptide synthesis: Peptide chains were extended on a Tentagel R RAM resin (0.20 mmol g^{-1}). For CF experiments, a modular CF apparatus was assembled, consisting of a cylindrical PEEK column (with internal dimensions of 250×4 mm) filled with the resin-loaded amino acid (350 mg), a semi-preparative pump (JASCO PU-4086), an HPLC autosampler (JASCO AS-4150), a column oven (JASCO CO-4060), two line-selecting valve units (JASCO HV-4380), and a backpressure regulator. A coupling mixture, consisting of 1.5 equivalents of Fmoc-protected β-amino acid and 1.5 equivalents of OxymaPure as coupling reagent dissolved in DMF and 1.5 equivalents of DIC, was mixed by the autosampler. The coupling mixture has been prepared immediately before the coupling reaction. Coupling reactions were carried out at the optimized reaction conditions, 85 bar pressure, 65 °C temperature, and 0.2 mL min^{-1} flow rate. For Fmoc deprotection the solution of 2 mL of 2% DBU 2% piperidine in DMF has been used. Between two chemical steps DMF was used for washing for 5 min.

NMR experiments:

NMR measurements for signal assiigment were carried out on a Bruker Avance III 500 MHz spectrometer equipped with a cryo probe head. Peptide samples (4 mM) were prepared in H_2O/D_2O 90:10 v/v, DMSO-d_6 or CD_3OH and transferred into 5 mm NMR sample tubes. For the ROESY spinlock, a mixing time of 300 ms was used; the number of scans was 16 and roesyesgpph or roesyph.2 (DMSO-d_6) pulse sequence was applied. The TOCSY measurement was performed with the mlevesgpph or mlevph (DMSO-d_6) sequence, with a mixing time of 150 ms; the number of scans was 16. For all 2D spectra, 4k time domain points and 512 increments were applied. T2 relaxation experiments were carried out by cpmg_esgp2d or cpmg (DMSO-d_6) pulse sequence; the relaxation delays were incremented in the following order: 1, 2, 4, 8, 16, 32, 64, 128, 256, 512, and 1024 ms.

The NH/ND exchange was recorded on a Varian Mercury 400 spectrometer equipped with ATB PFG probe head. The samples were prepared in 4 mM concentration in CD_3OD and

transferred into standard 5 mm NMR sample tubes. The recording was started 10 minutes after complete solubilization. The ^{1}H spectra were recorded using 64 scans with 45 degree pulse and 5 s relaxation delay.

CD measurements:

CD spectra were measured on a Jasco J-1500 spectropolarimeter at 25 °C in a 0.1 cm path length regtangular quartz cuvette (Hellma, Plainview, NY) in a continuous scanning mode between 190 and 250 nm at a rate of 50 nm min^{-1}, with a data pitch of 0.5 nm, a response time of 4 s, a 1 nm bandwidth, and 3 times accumulation for each sample. The baseline spectrum recorded with the solvent was subtracted from the raw data. The concentration of the sample solutions in ultrapure water and PBS was 1 mM. Molar circular dichroism is given in mol^{-1} cm^{-1}.

ATR-FTIR measurement:

A Varian 2000 FTIR Scimitar spectrometer (Varian Inc., Palo Alto, CA) was used for FTIR spectroscopic measurements. The spectrometer is fitted with a liquid nitrogen-cooled mercury-cadmium-telluride (MCT) detector with a "Golden Gate" single reflection diamond ATR accessory (Specac Ltd., Orpington, U.K.). On the diamond ATR surface, 3 µL of the sample was mounted and the spectrum was accumulated (2 cm^{-1} resolution and 64 scans) for the dry film after gradual evaporation of the buffered solvent under ambient conditions. ATR correction for every data acquisition, buffer subtraction and baseline corrections were performed. The GRAMS/32 software package (Galactic Inc.) was used for all spectral manipulations.

TEM measurement:

The peptides were dissolved in water to a concentration of 1 mM, and the solution was sonicated for 5 min. Drops of 5 µL of solutions were placed onto Formvar-coated 200-mesh copper grids (Ted Pella Inc, USA) and dried in the air at 25 °C for 10 min. Specimens were studied with a MORGAGNI 268D transmission electron microscope (FEI, Eindhoven, The

Netherlands) operated at 80 kV and equipped with a Quemesa 11-megapixel bottom-mounted CCD camera (Emsis GmbH, Germany).

Model building and theoretical calculations

The structures were drawn with Schrödinger's Maestro 2D Sketcher, the hydrogen atoms were added and the structures were quickly optimised into 3D geometry. A conformational search for each compound was completed with the MacroModel package "Energy minimization" option using the OPLS4 force field with "no solvent" option with the default settings. In the case of dimer **3**, in order to maintain the interaction between the two chains, "frozen" atom constraints were applied on the corresponding hydrogen and oxygen atoms participating in the H-bonds. The output structures of the minimization of each compound were used as initial geometries for further calculations.

The Jaguar geometry optimization calculations were completed in two steps. First, the Restricted Hartree-Fock (RHF) method was applied with 3-21G basis set in a vacuum. Second, Density Functional Theory (DFT) method (B3LYP-D3 functional) was applied with 6-311G** basis set in vacuum. In the case of dimer **3**, implicit water was necessary in order to maintain chain-like conformation, and the Polarizable continuum model (PCM) solvation model was applied.

Software packages as follows were used: Maestro (v13.8): Release 2023-4, Schrödinger, LLC, New York, NY, 2023; MacroModel (v14.2): Release 2023-4, Schrödinger, LLC, New York, NY, 2023; Jaguar (v12.2): Release 2023-4, Schrödinger, LLC, New York, NY, 2023.

Peptide synthesis – HPLC-MS data

Acquisition Parameter

Ion Source Type	ESI	Ion Polarity	Positive	Alternating Ion Polarity	off
Mass Range Mode	Std/Enhanced	Scan Begin	100 m/z	Scan End	2800 m/z
Capillary Exit	220.0 Volt	Skimmer	40.0 Volt	Trap Drive	78.0
Accumulation Time	3165 µs	Averages	3 Spectra	Auto MS/MS	off

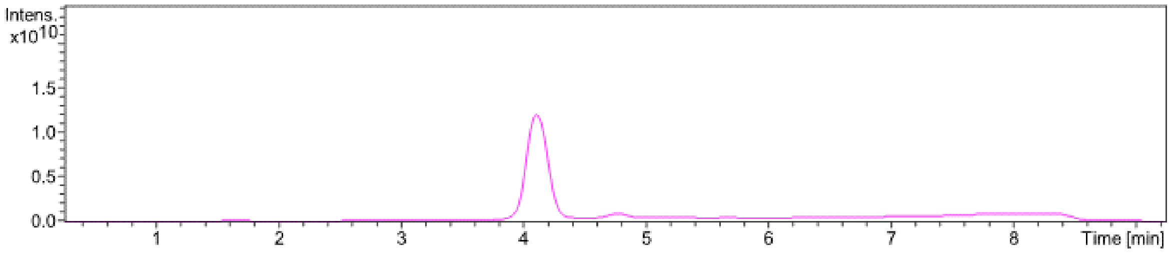

+MS, 4.0-4.3min #(199-216)

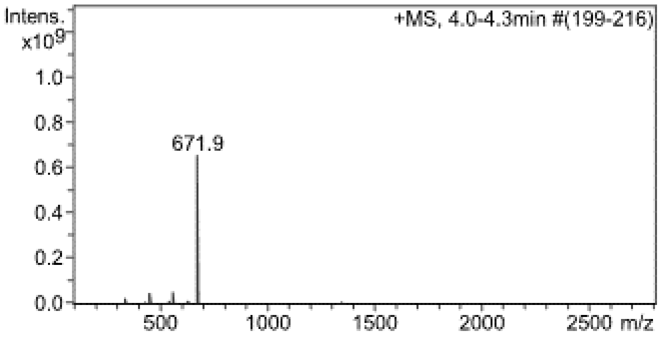

#	m/z	I
1	338.8	26564088
2	449.8	45326788
3	542.9	14123194
4	560.9	53682044
5	561.9	12018386
6	627.9	12122475
7	671.0	23652508
8	671.9	654528512
9	672.9	279078048
10	673.9	78515432

Figure S1. HPLC-MS for compound **1**

Acquisition Parameter

Ion Source Type	ESI	Ion Polarity	Positive	Alternating Ion Polarity	off
Mass Range Mode	Std/Enhanced	Scan Begin	100 m/z	Scan End	2800 m/z
Capillary Exit	220.0 Volt	Skimmer	40.0 Volt	Trap Drive	78.0
Accumulation Time	2628 µs	Averages	3 Spectra	Auto MS/MS	off

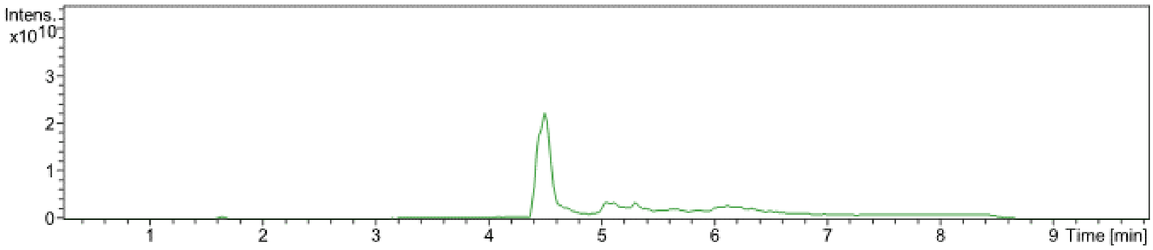

+MS, 4.4-4.6min #(224-232)

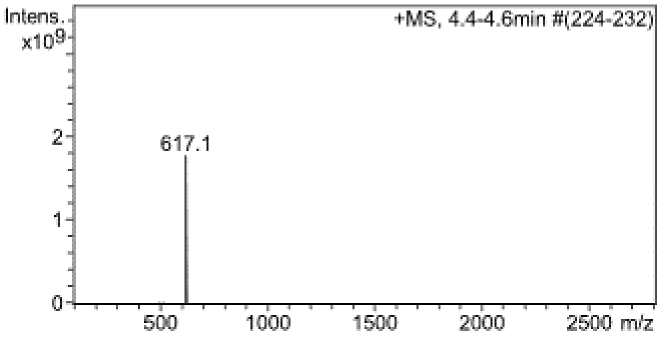

#	m/z	I
1	366.9	8813764
2	474.0	7822352
3	492.0	12788844
4	518.0	25209280
5	519.0	5668916
6	617.1	1777939072
7	618.0	734995584
8	619.0	184255936
9	620.0	17926652
10	688.0	7393353

Figure S2. HPLC-MS for compound **2**

Acquisition Parameter

Ion Source Type	ESI	Ion Polarity	Positive	Alternating Ion Polarity	off
Mass Range Mode	Std/Enhanced	Scan Begin	100 m/z	Scan End	2800 m/z
Capillary Exit	220.0 Volt	Skimmer	40.0 Volt	Trap Drive	78.0
Accumulation Time	3111 µs	Averages	3 Spectra	Auto MS/MS	off

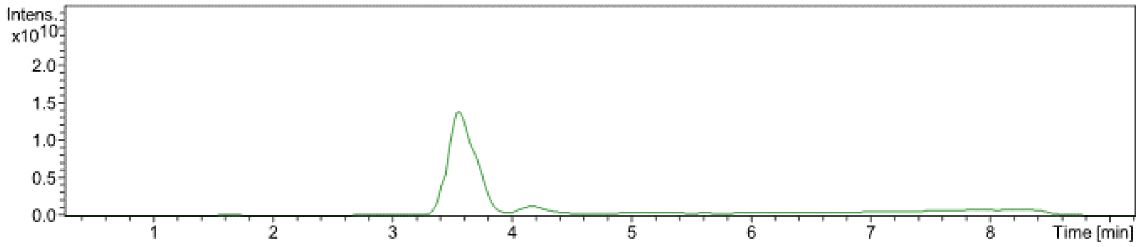

+MS, 3.4-3.8min #(167-188)

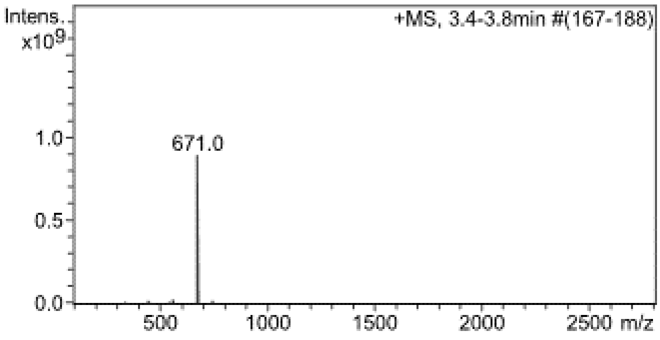

#	m/z	I
1	448.8	15830007
2	541.9	11037777
3	559.9	26272308
4	560.9	8342897
5	671.0	894425472
6	671.9	405727712
7	673.0	108604216
8	673.9	16292975
9	741.9	20061102
10	743.0	8356744

Figure S3. HPLC-MS for compound **3**

NMR results

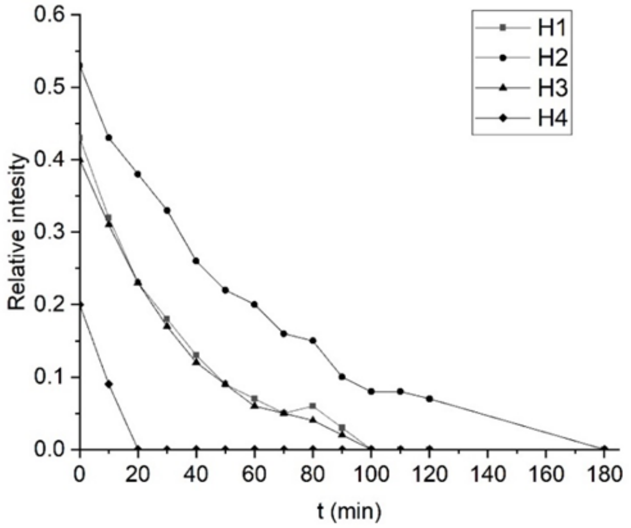

Figure S4. Time dependence of the NH/ND exchange for 4 mM solutions of 1 in CD_3OD

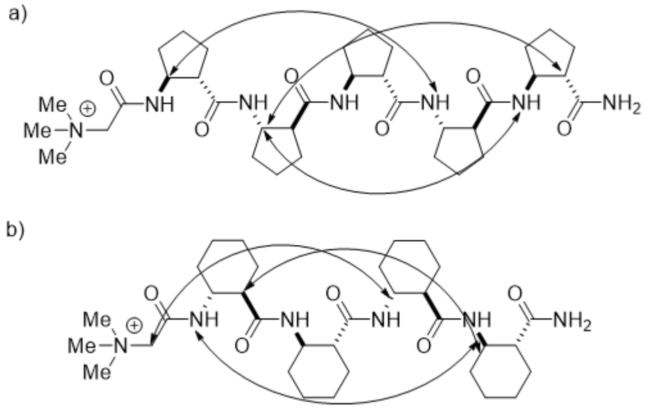

Figure S5. Long-range NOE interactions for 1 (a) in DMSO-d_6, and for 2 (b) found in DMSO-d_6, CD_3OH or water (90% H_2O + 10% D_2O)

Figure S6. Assignment of backbone Hs of compound **1** in CD_3OH

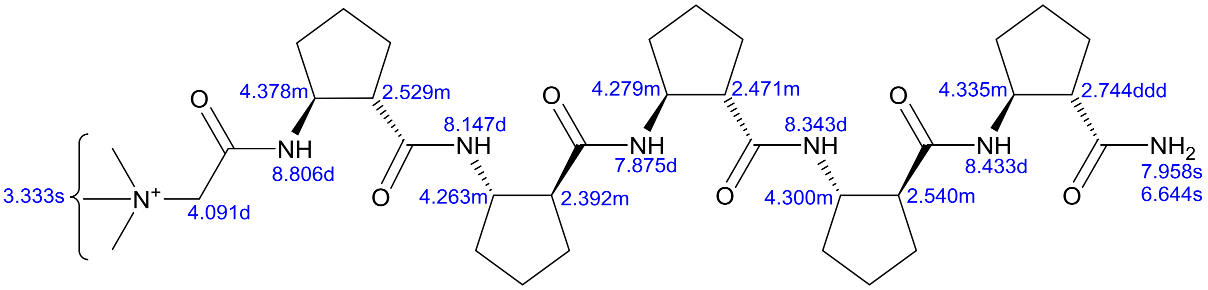

Figure S7. ^{1}H NMR spectrum of compound **1** in CD_3OH

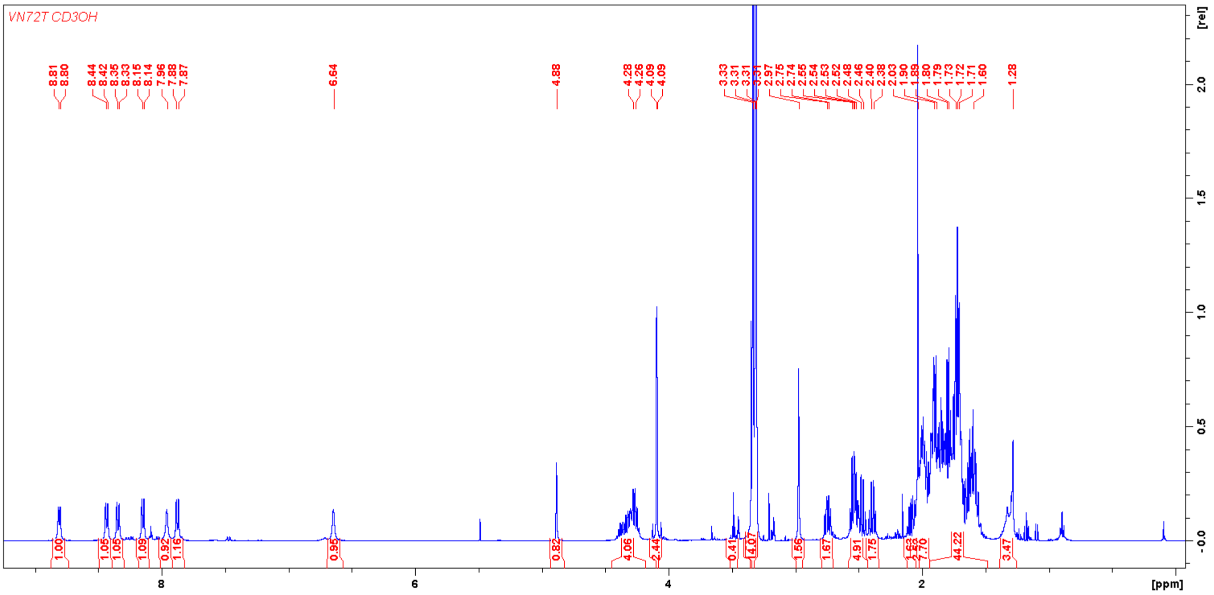

Figure S8. TOCSY NMR spectrum of compound **1** in CD_3OH

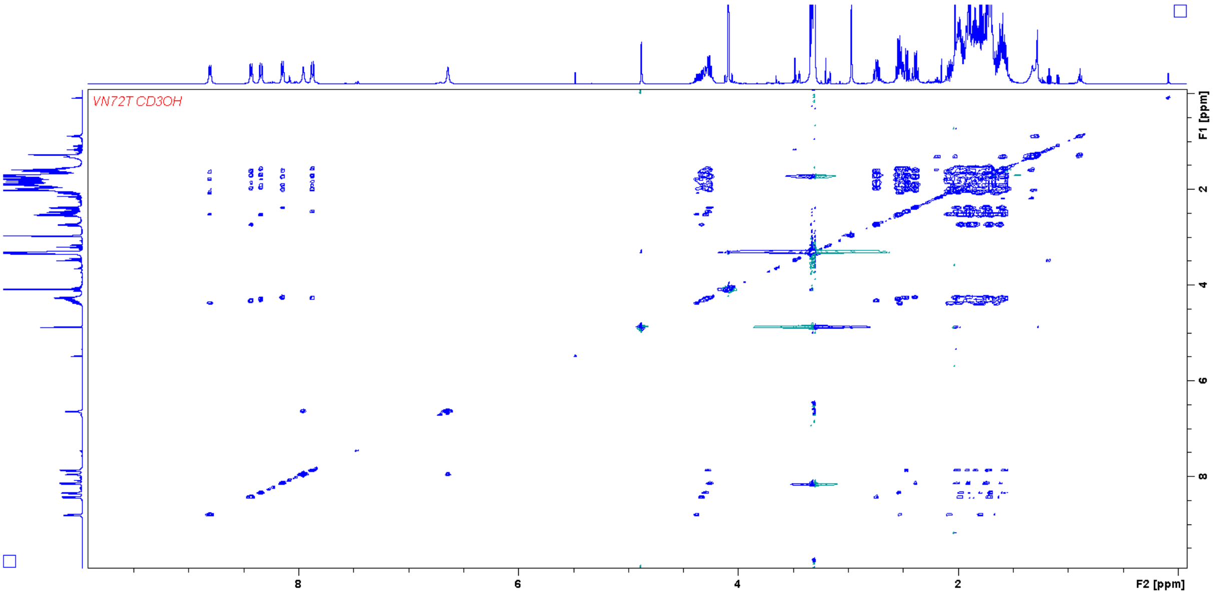

Figure S9. ROESY NMR spectrum of compound **1** in CD_3OH

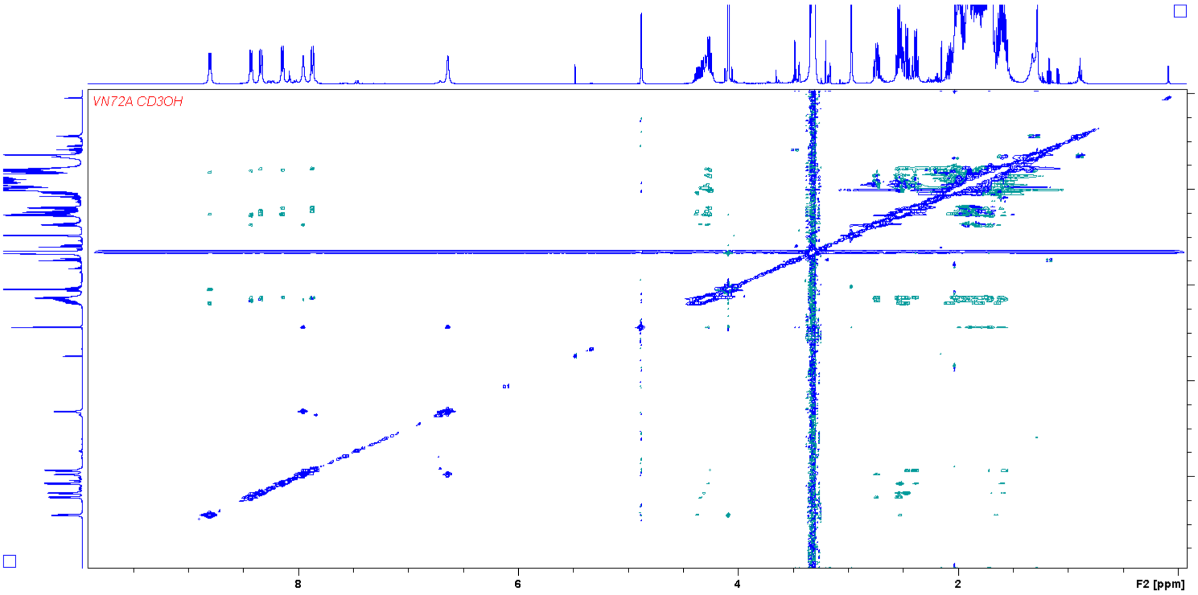

Figure S10. Assignment of backbone Hs of compound **1** in H_2O/D_2O 9:1

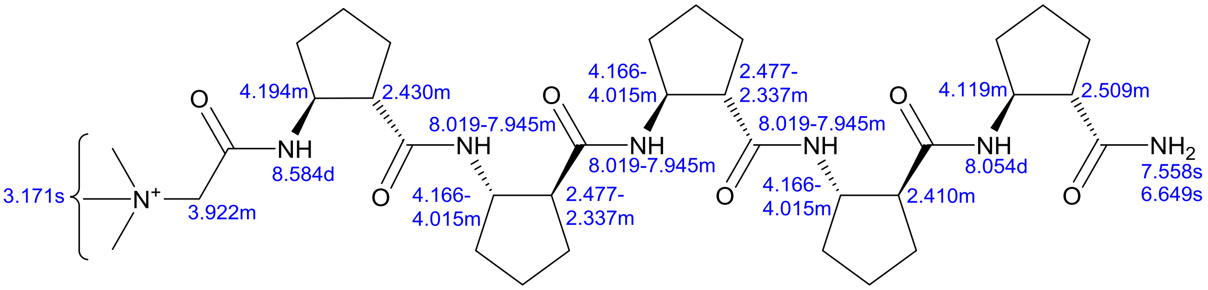

Figure S11. ^{1}H NMR spectrum of compound **1** in H_2O/D_2O 9:1

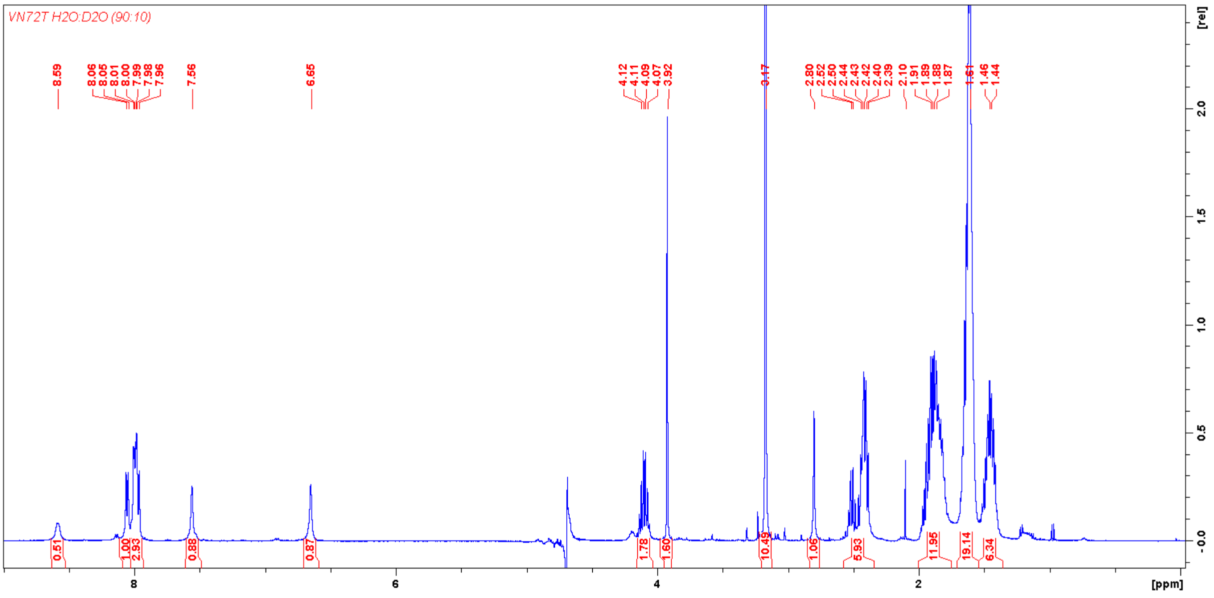

Figure S12. TOCSY NMR spectrum of compound **1** in H_2O/D_2O 9:1

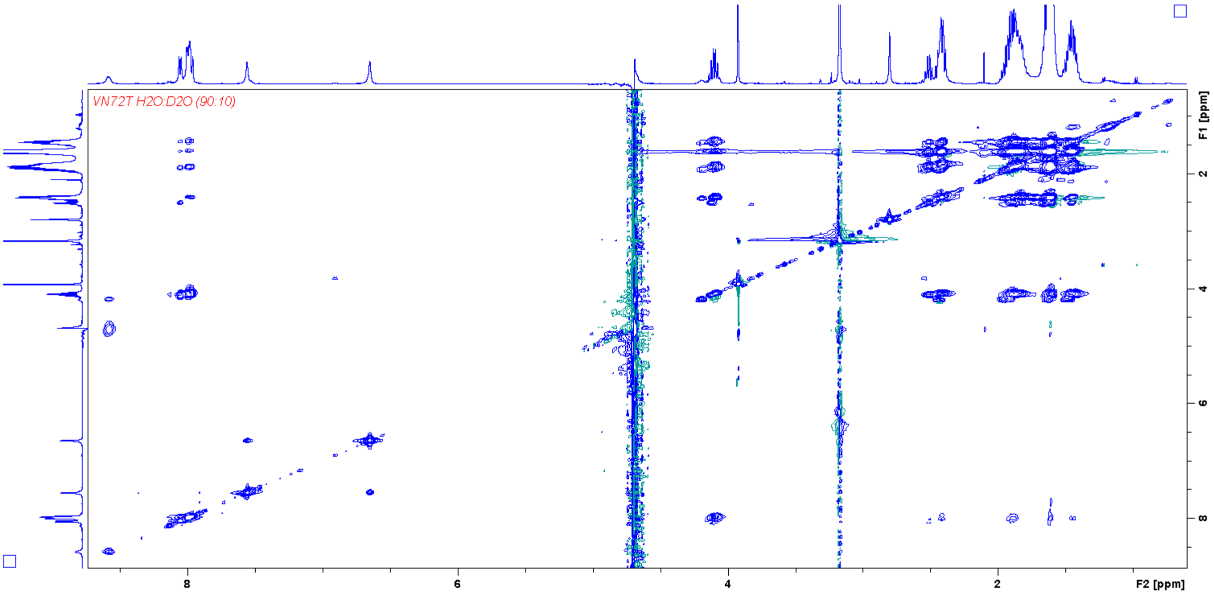

Figure S13. ROESY NMR spectrum of compound **1** in H_2O/D_2O 9:1

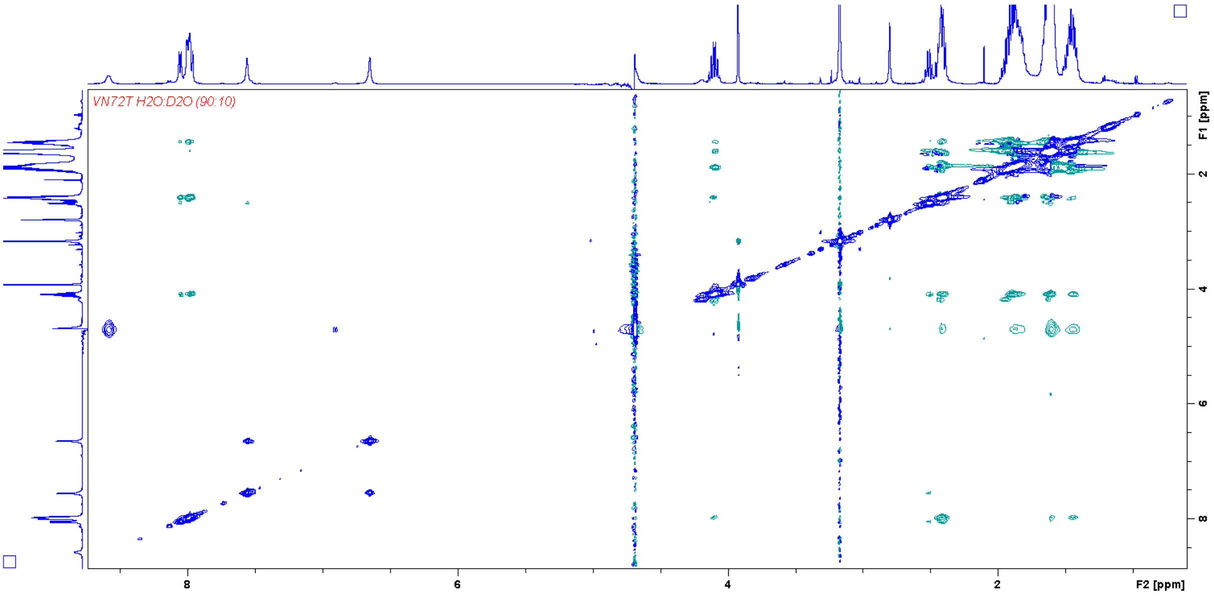

Figure S14. Assignment of backbone Hs of compound **1** in DMSO-d_6

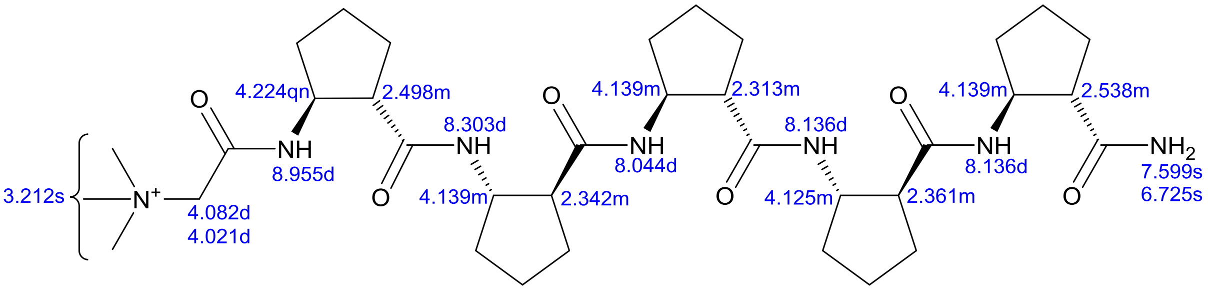

Figure S15. ^{1}H NMR spectrum of compound **1** in DMSO-d_6

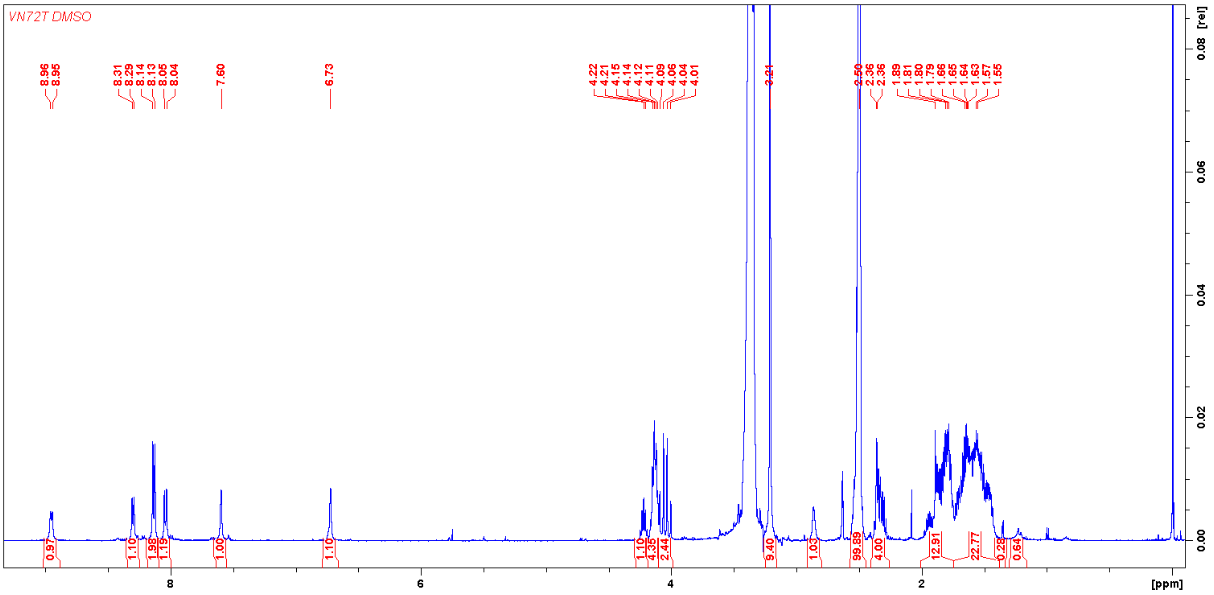

Figure S16. TOCSY NMR spectrum of compound **1** in DMSO-d_6

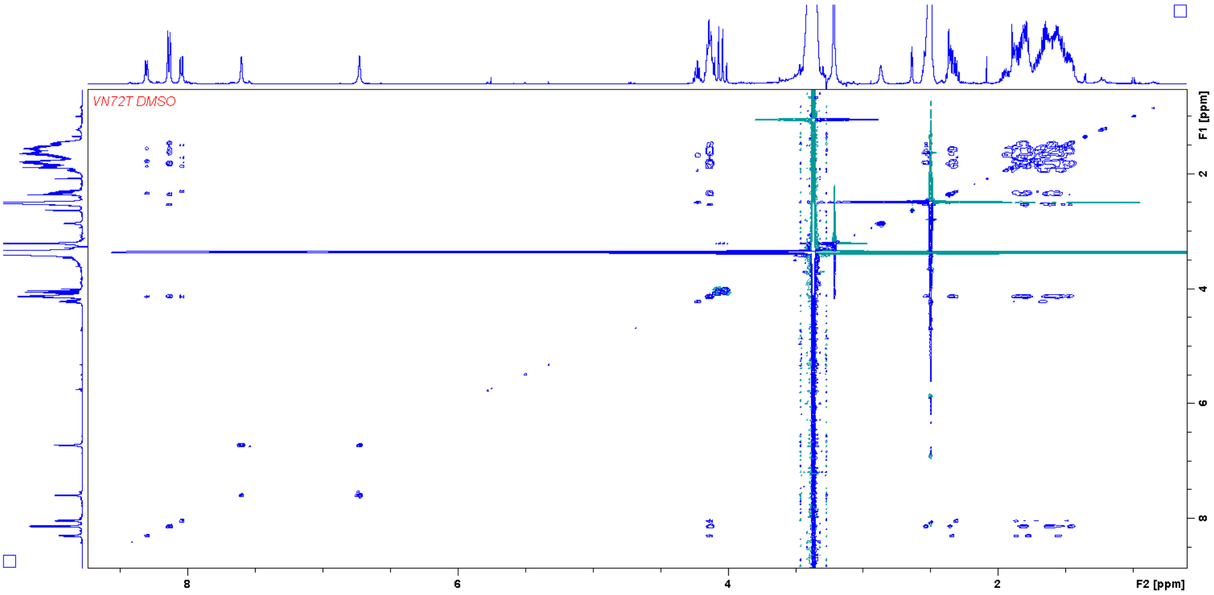

Figure S17. ROESY NMR spectrum of compound **1** in DMSO-d_6

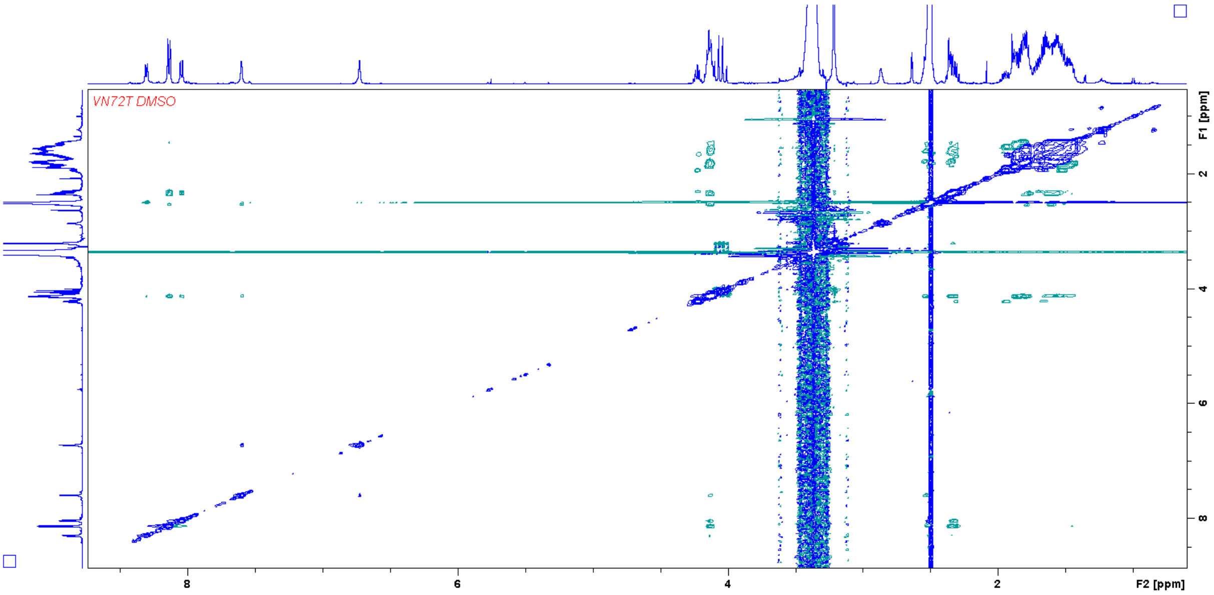

Figure S18. Assignment of backbone Hs of compound **2** in CD_3OH

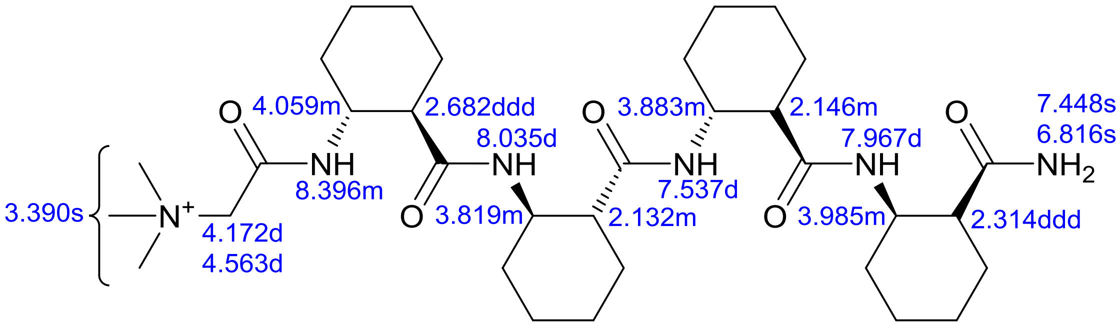

Figure S19. ^{1}H NMR spectrum of compound **2** in CD_3OH

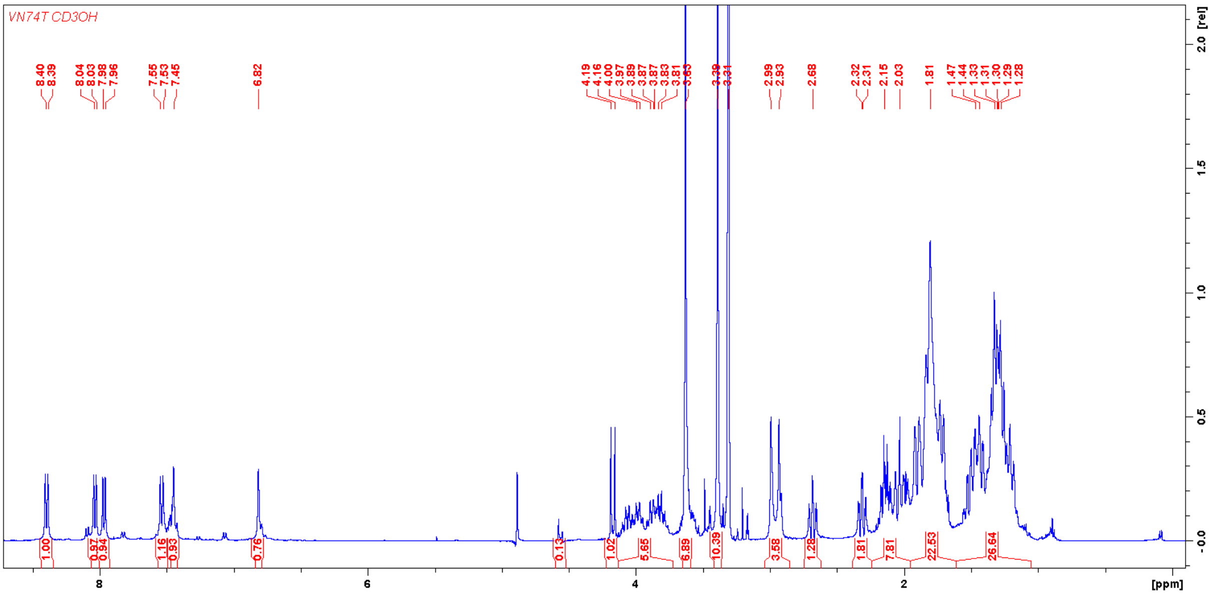

Figure S20. TOCSY NMR spectrum of compound **2** in CD_3OH

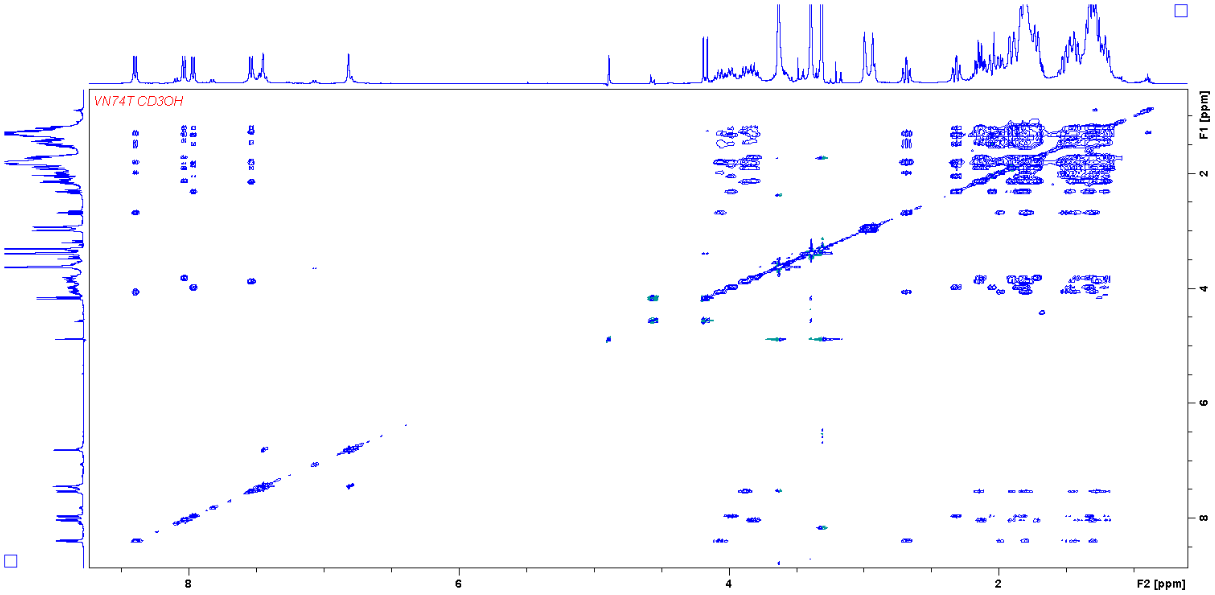

Figure S21. ROESY NMR spectrum of compound **2** in CD_3OH

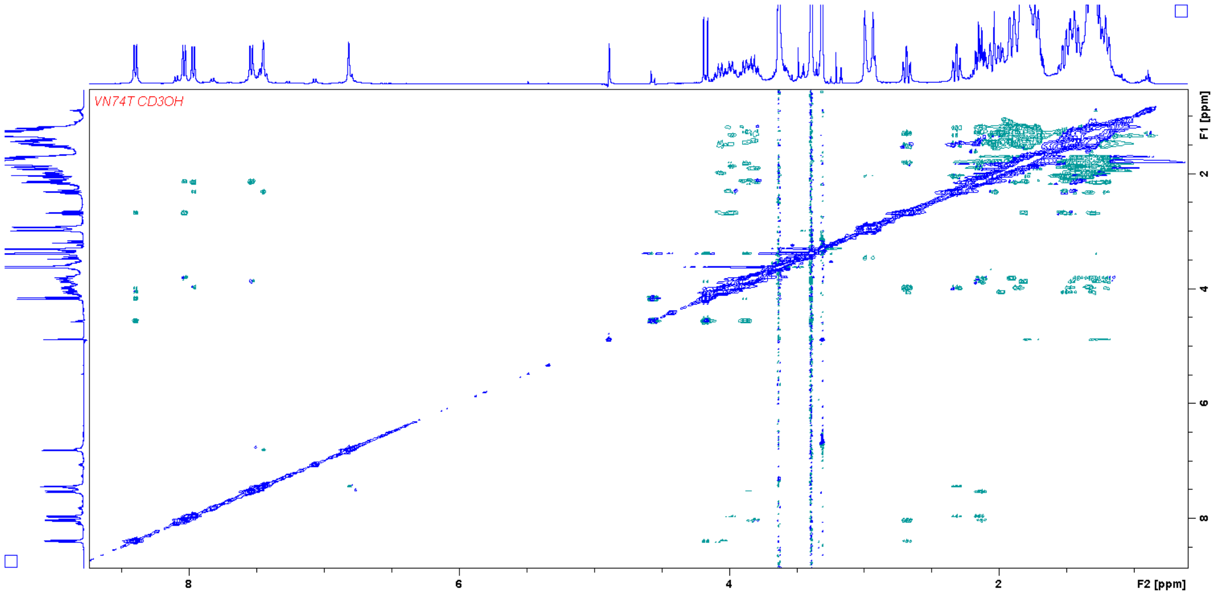

Figure S22. Assignment of backbone Hs of compound **2** in H_2O/D_2O 9:1

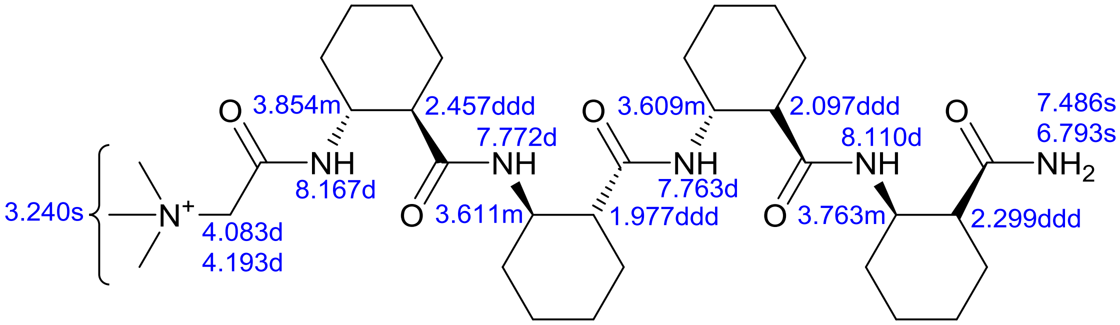

Figure S23. ^{1}H NMR spectrum of compound **2** in H_2O/D_2O 9:1

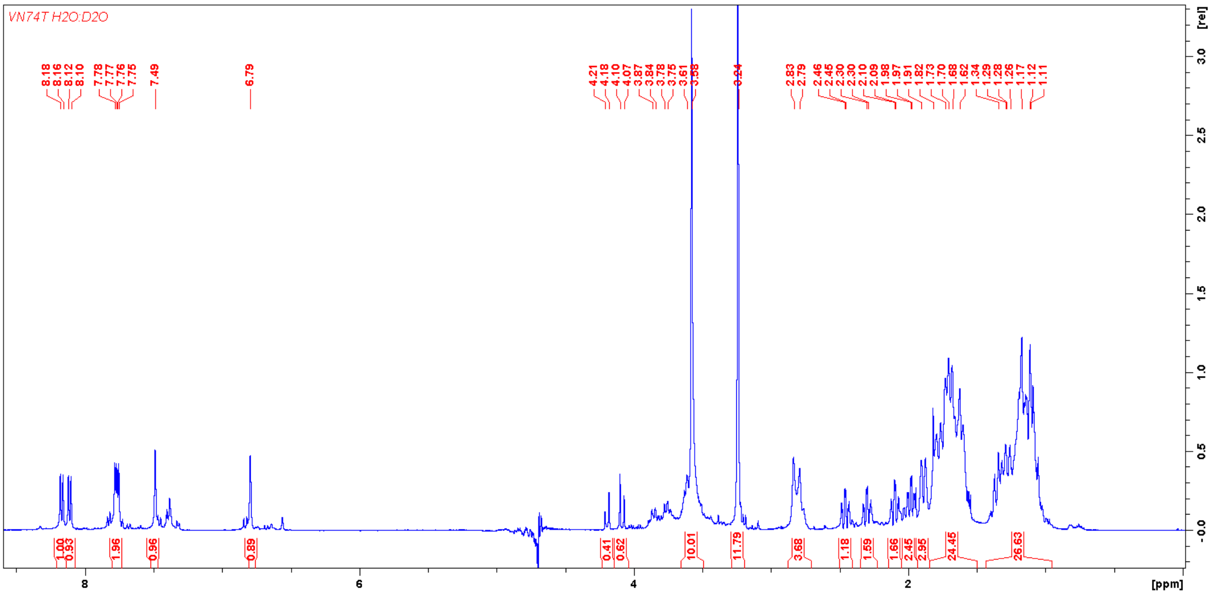

Figure S24. TOCSY NMR spectrum of compound **2** in H_2O/D_2O 9:1

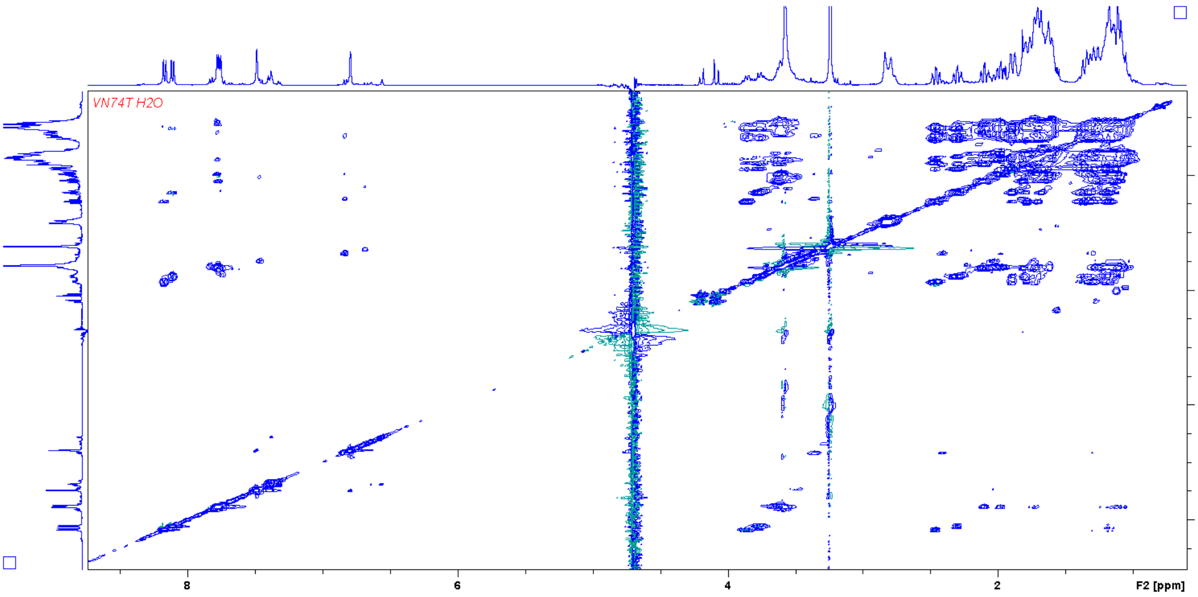

Figure S25. ROESY NMR spectrum of compound **2** in H_2O/D_2O 9:1

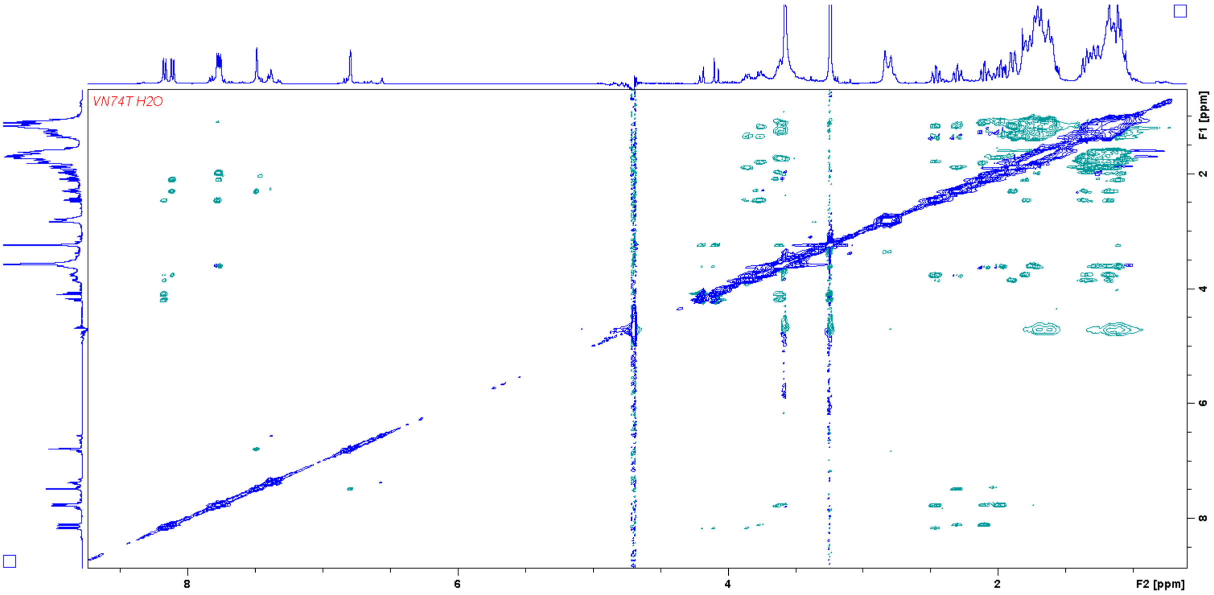

Figure S26. Assignment of backbone Hs of compound **2** in DMSO-d_6

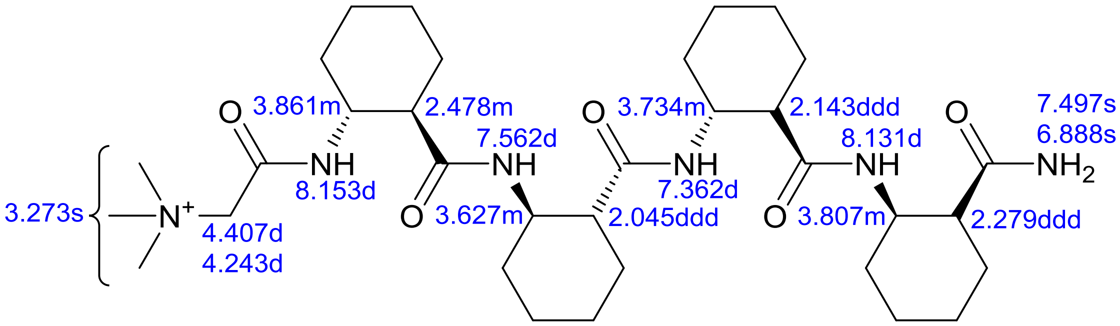

Figure S27. 1H NMR spectrum of compound **2** in DMSO-d_6

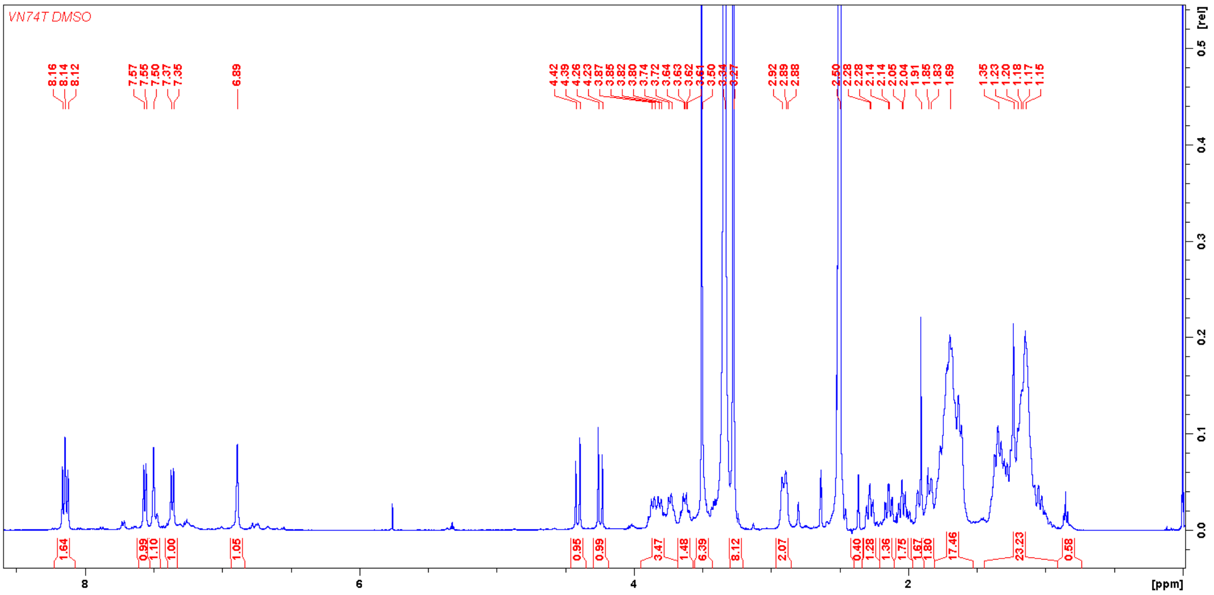

Figure S28. TOCSY NMR spectrum of compound **2** in DMSO-d_6

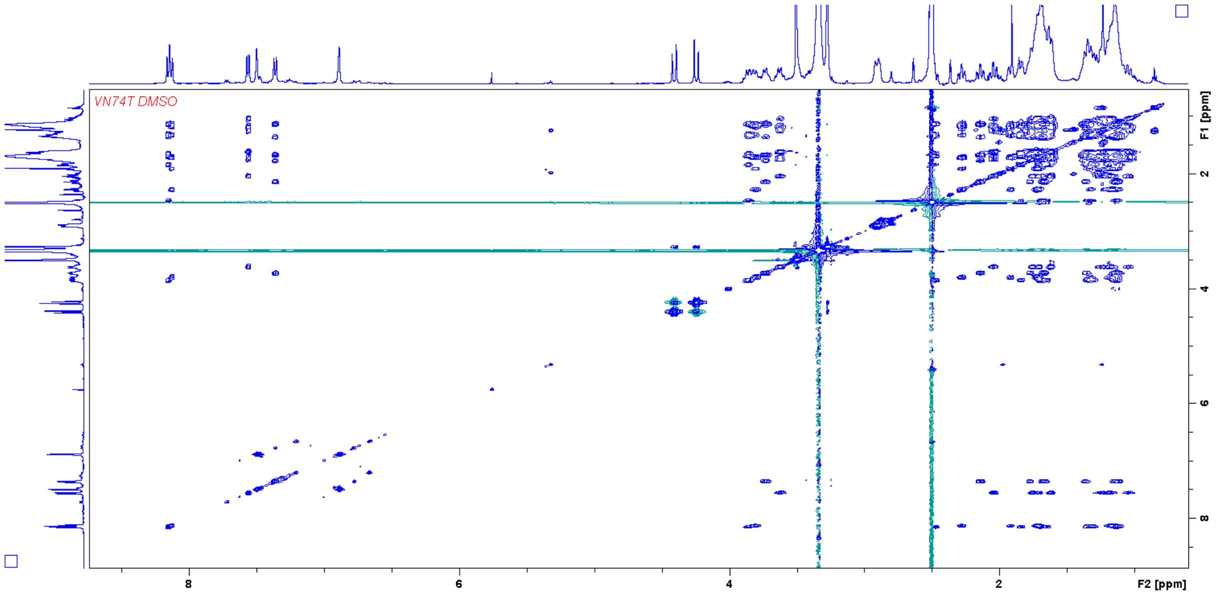

Figure S29. ROESY NMR spectrum of compound **2** in DMSO-d_6

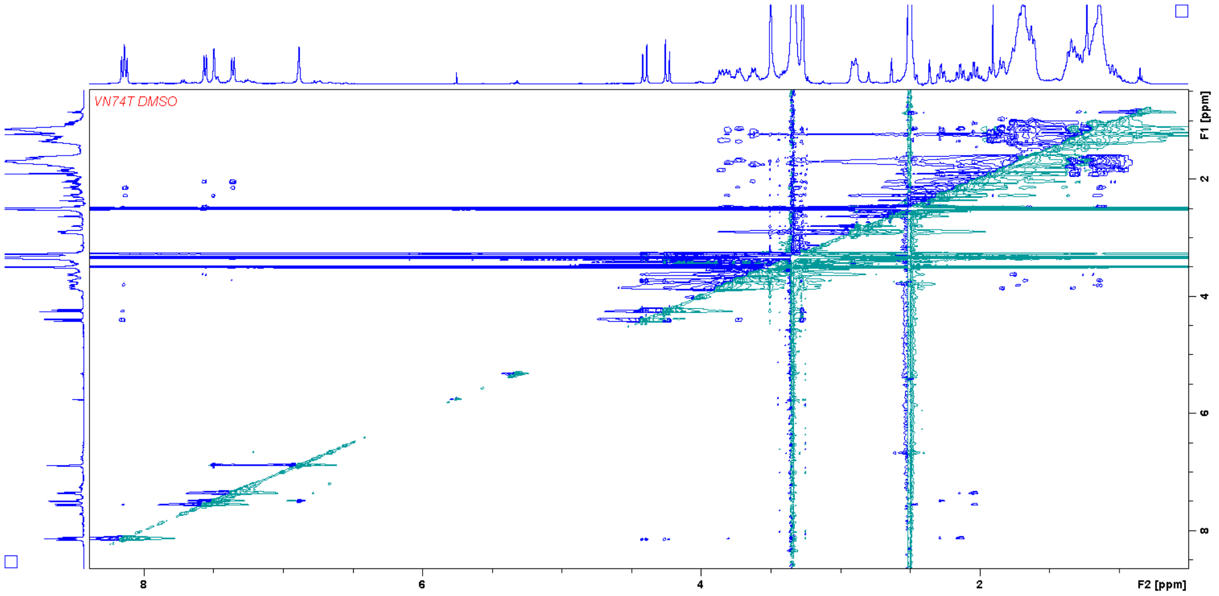

Figure S30. ^{1}H NMR spectrum of compound **3** in CD_3OH

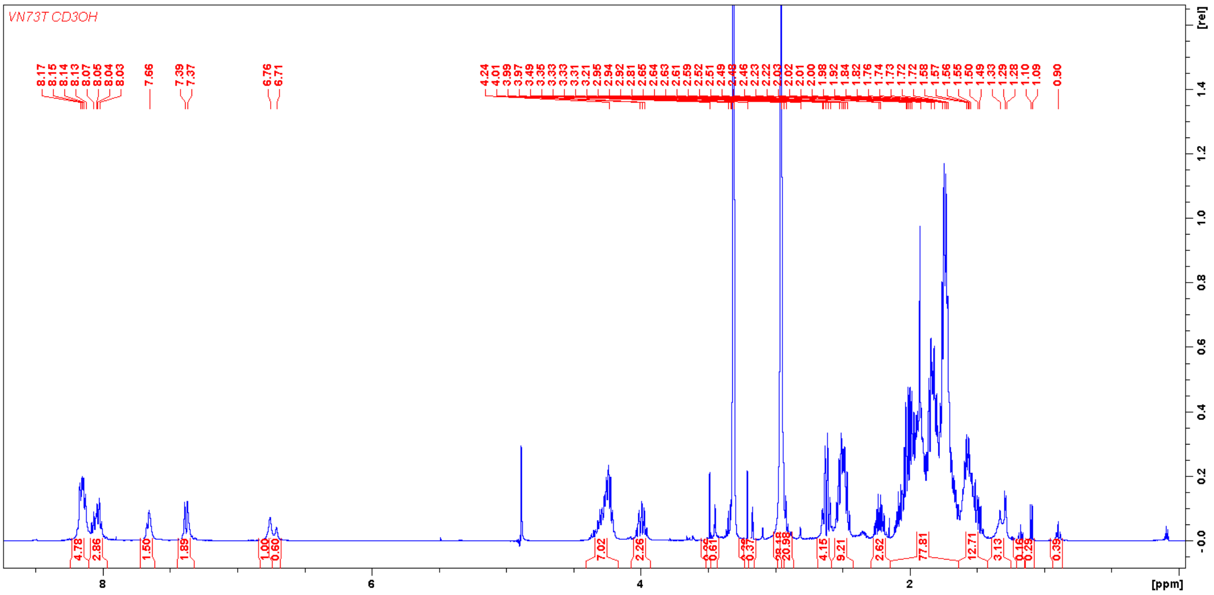

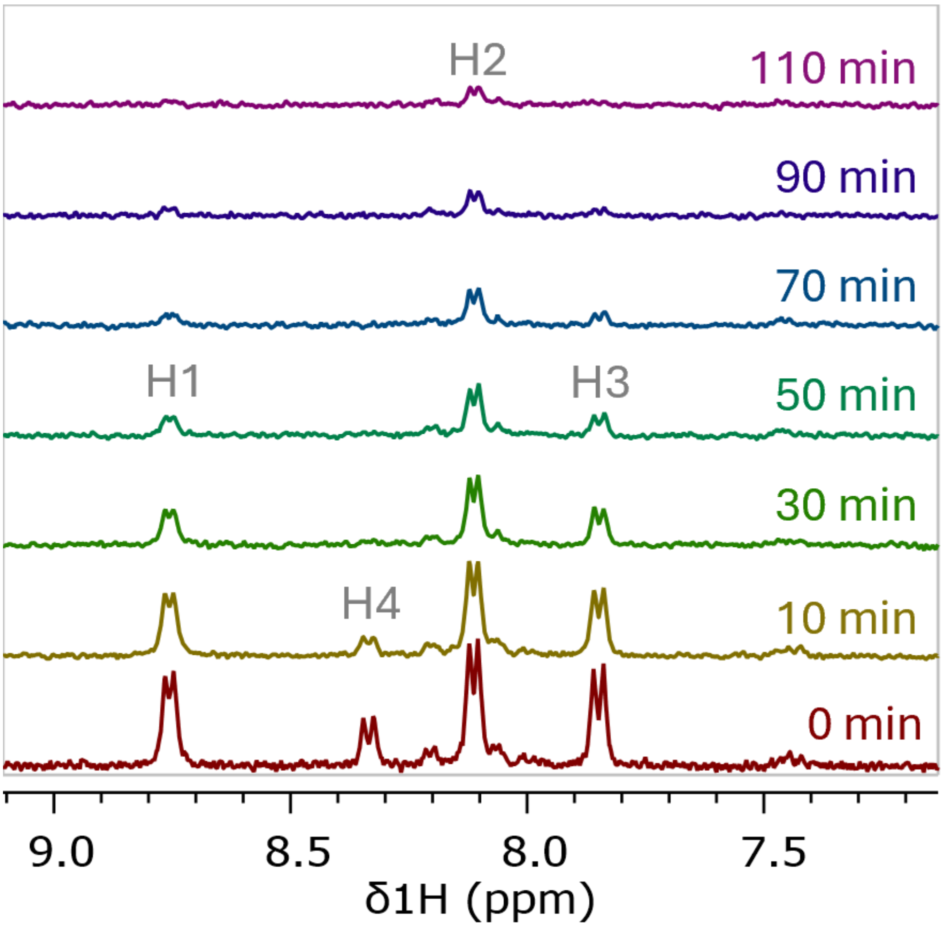

Figure S31. Time-dependent series of ^{1}H NMR spectra of compound **1**, showing the NH/ND exchange in CD_3OD at 297 K. The concentration of the sample was 4 mM.

TEM images

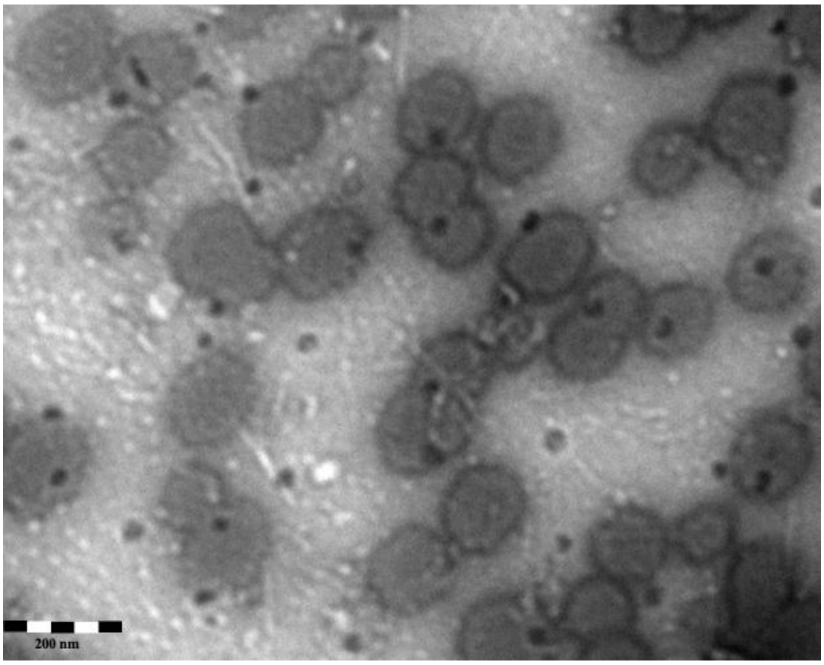

Figure S32. TEM image of vesicles observed after dissolution and sonication of 4 mM solutions of **1**.

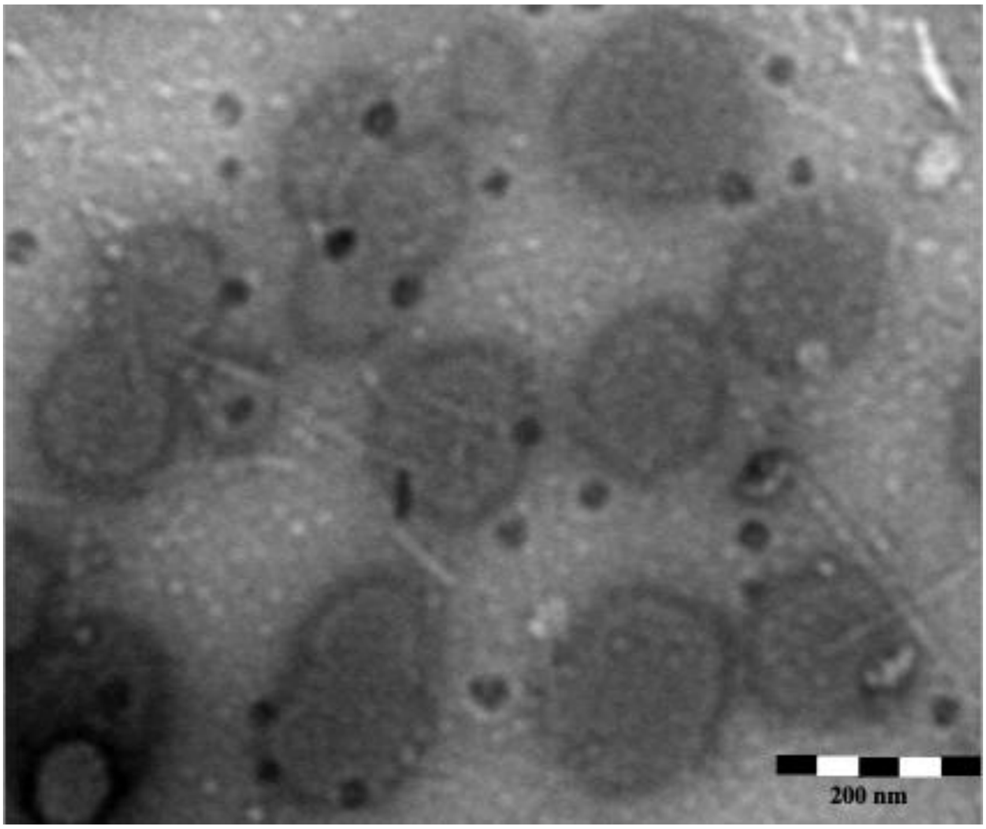

Figure S33. TEM image of vesicles observed after dissolution and sonication of 4 mM solutions of **1**.

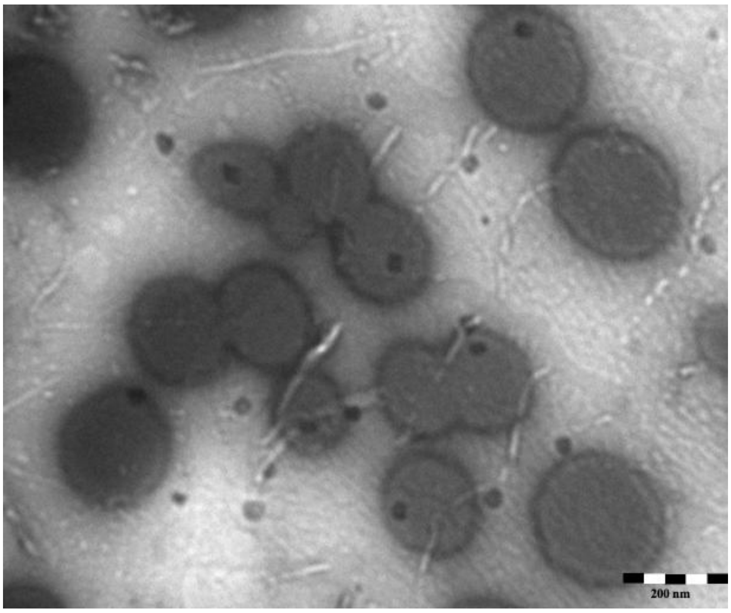

Figure S34. TEM image of vesicles observed after dissolution and sonication of 4 mM solutions of **1**.

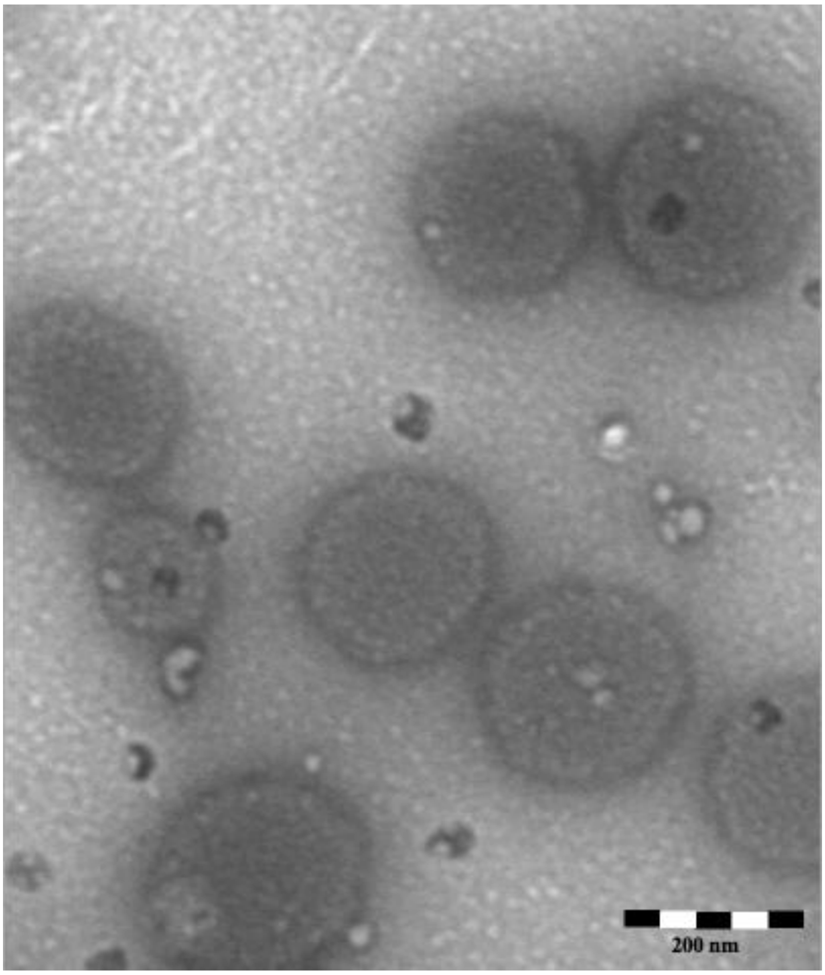

Figure S35. TEM image of vesicles observed after dissolution and sonication of 4 mM solutions of **2**.

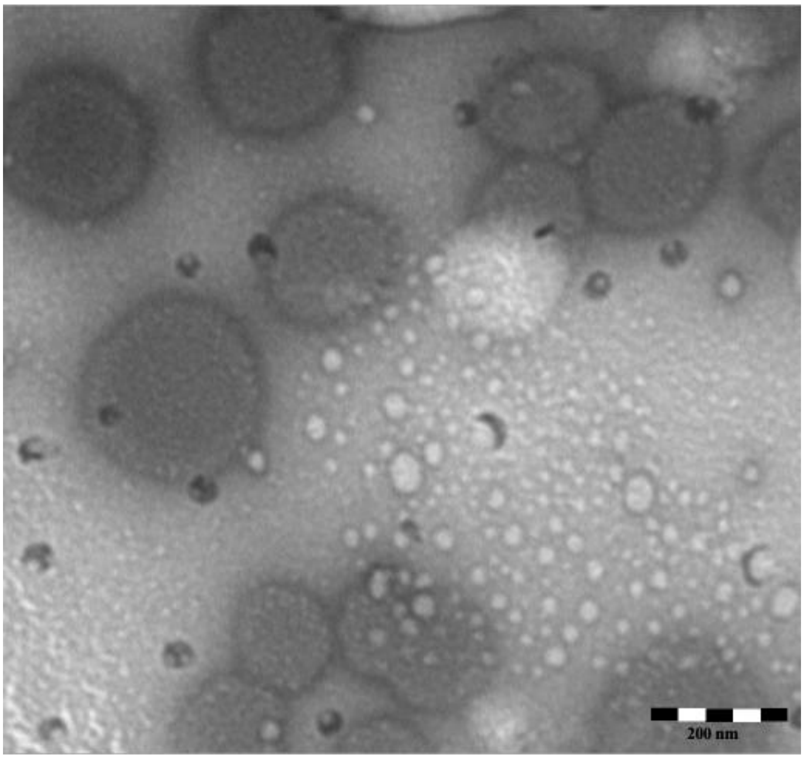

Figure S36. TEM image of vesicles observed after dissolution and sonication of 4 mM solutions of **2**.

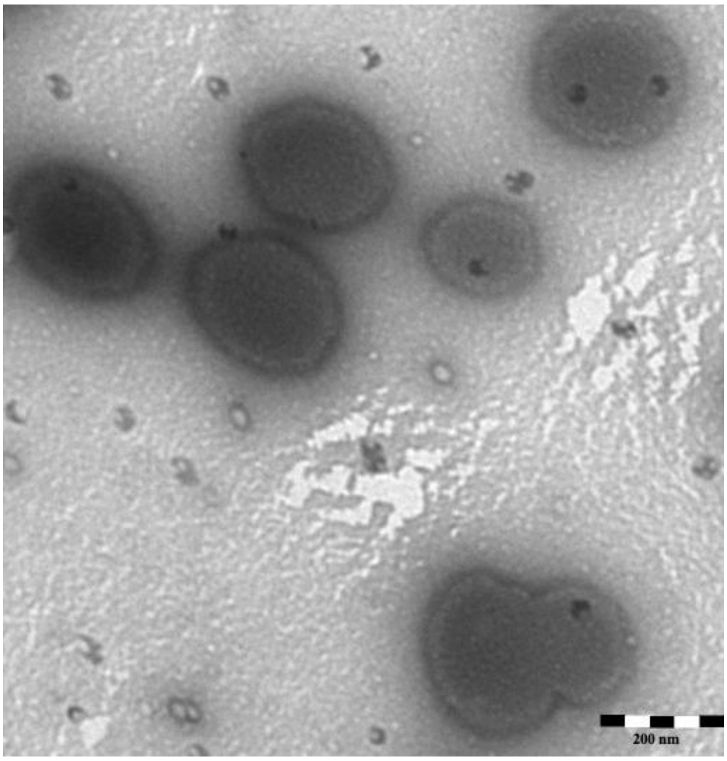

Figure S37. TEM image of vesicles observed after dissolution and sonication of 4 mM solutions of **2**.

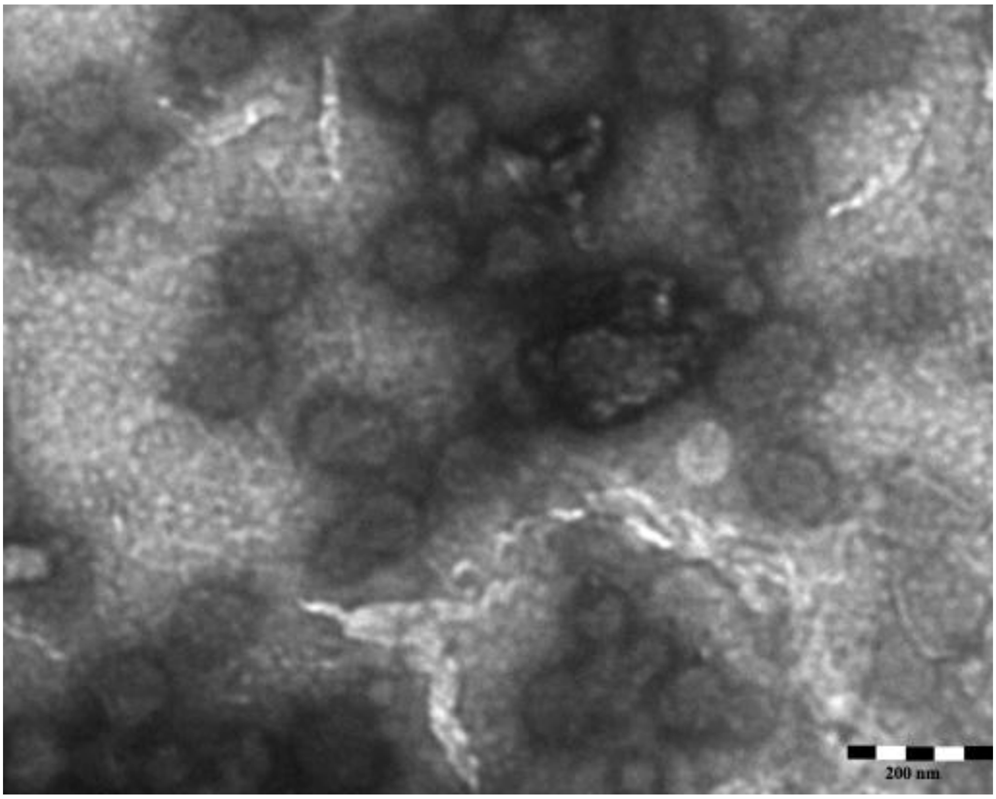

Figure S38. TEM image of vesicles observed after dissolution and 15 minute long sonication of 4 mM solutions of **2**.

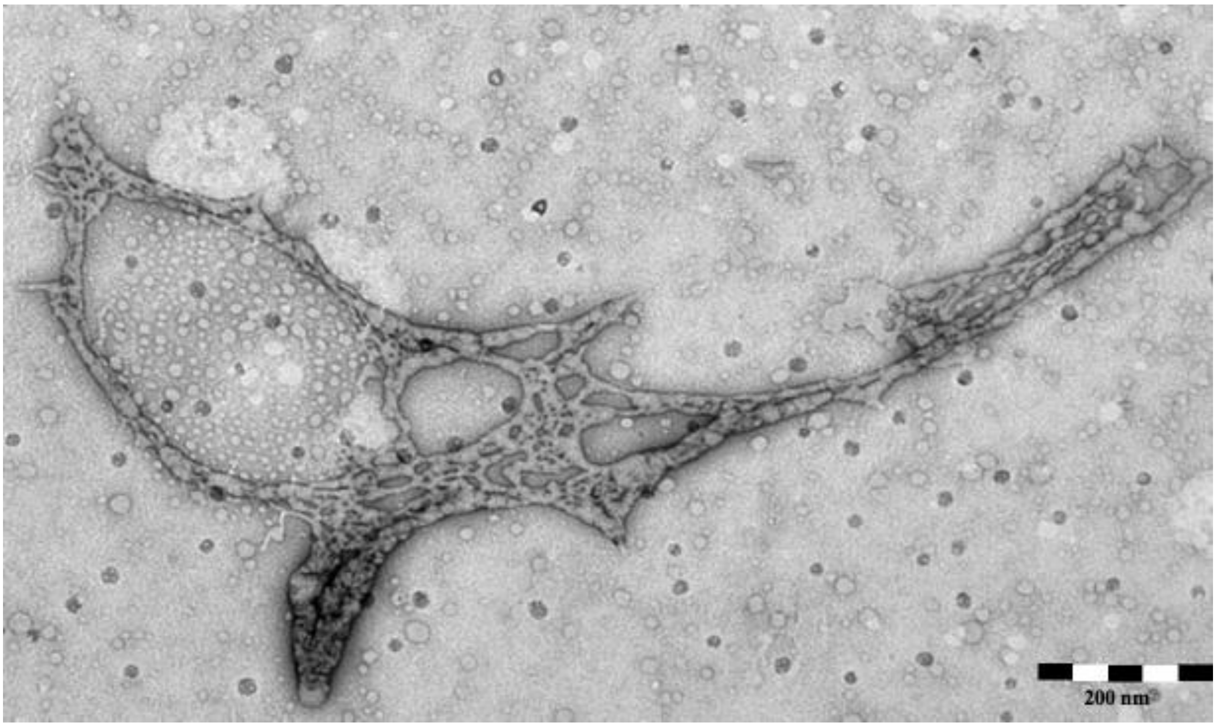

Figure S39. TEM image of vesicles observed after dissolution and sonication of 4 mM solutions of **3**.

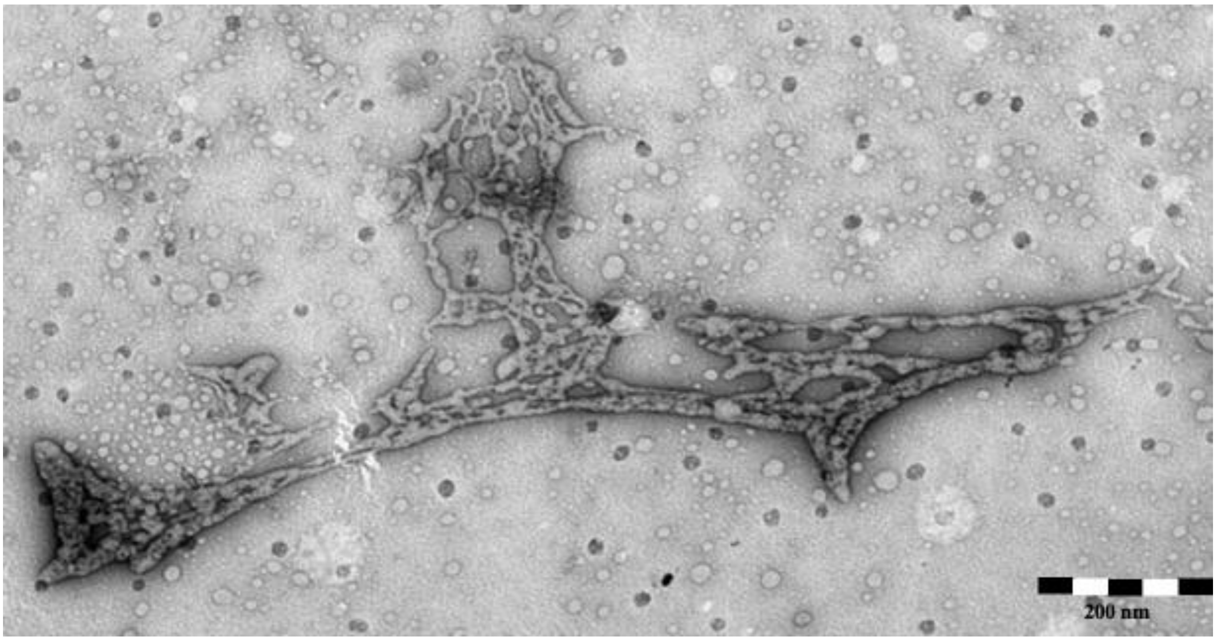

Figure S40. TEM image of vesicles observed after dissolution and sonication of 4 mM solutions of **3**.

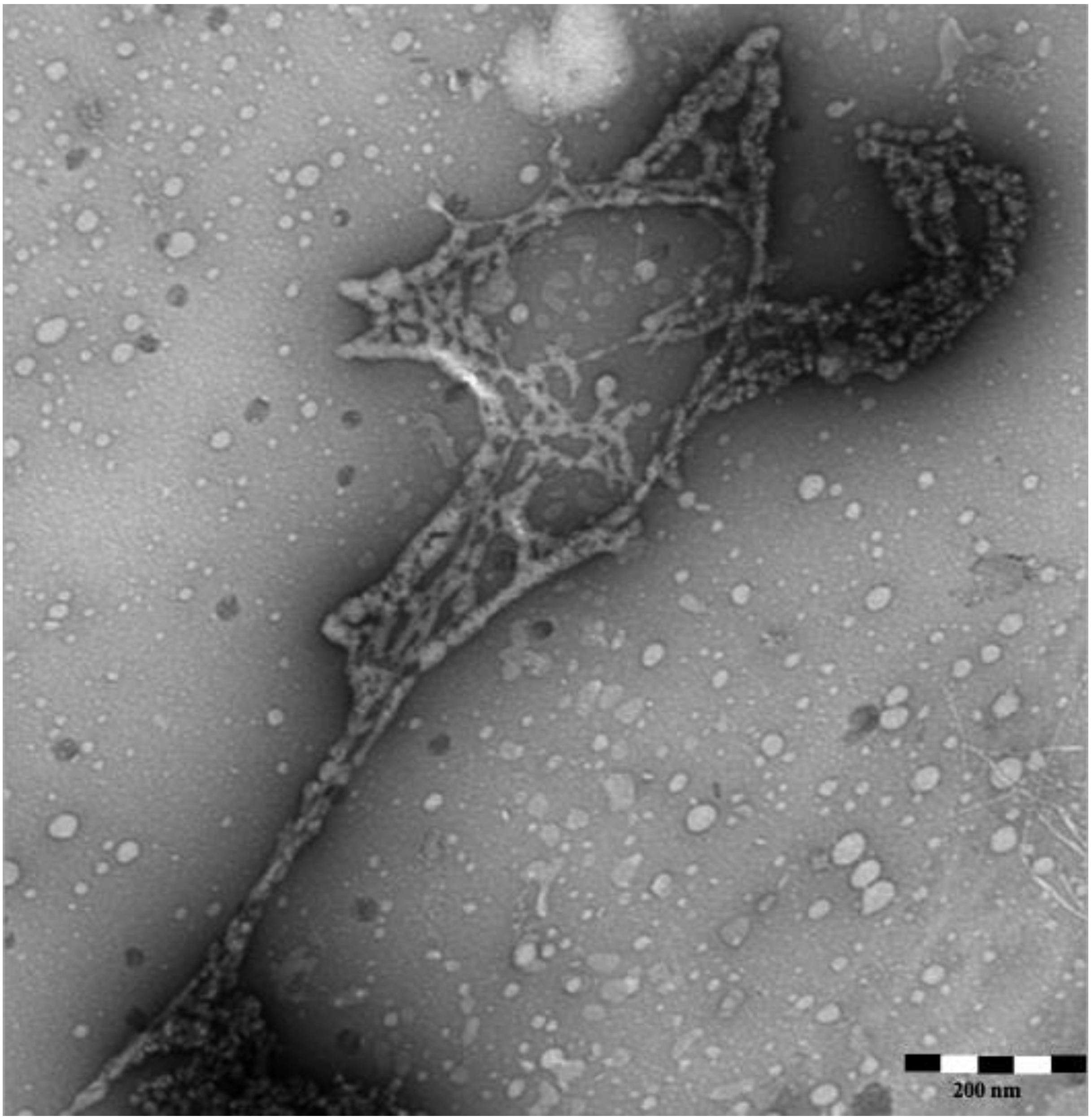

Figure S41. TEM image of vesicles observed after dissolution and sonication of 4 mM solutions of **3**.

HR-MS data

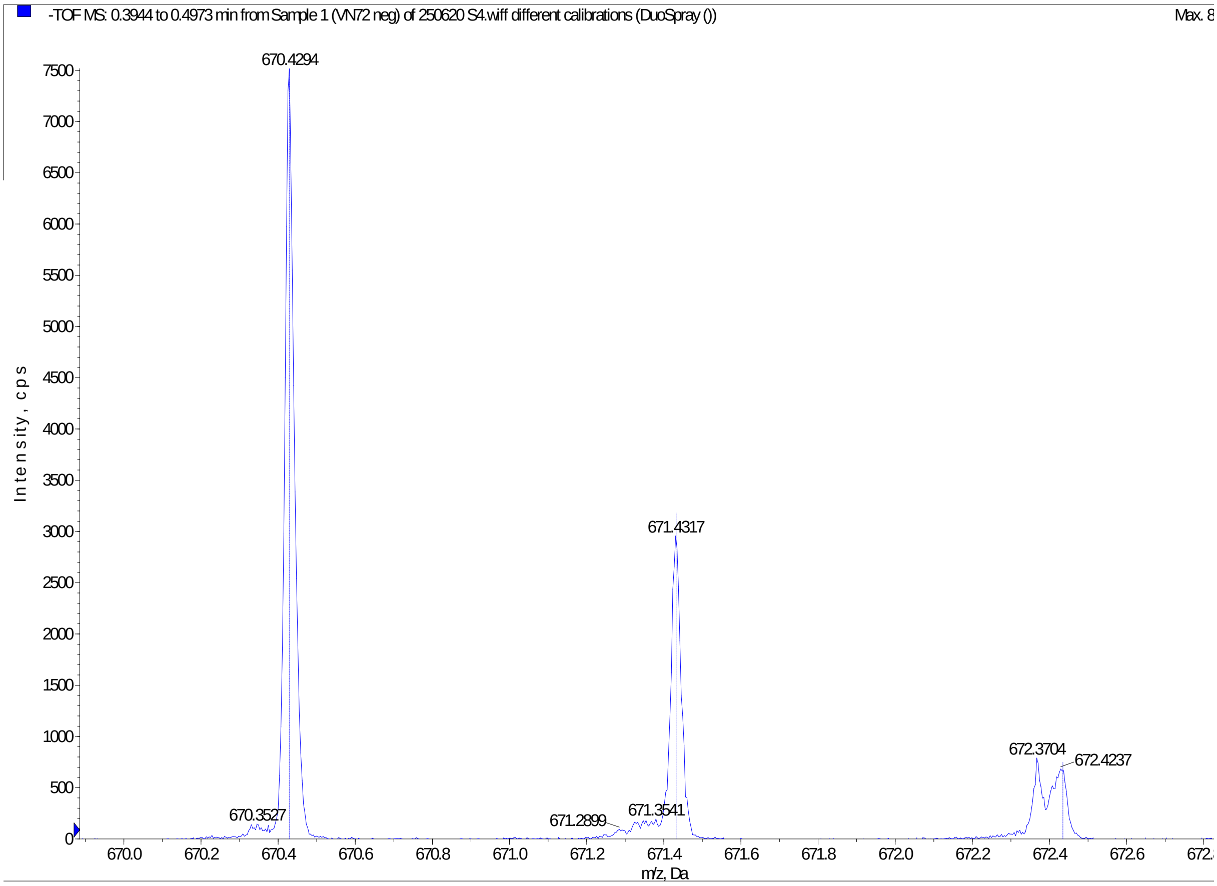

Figure S42. HR-MS spectrum of **1.**

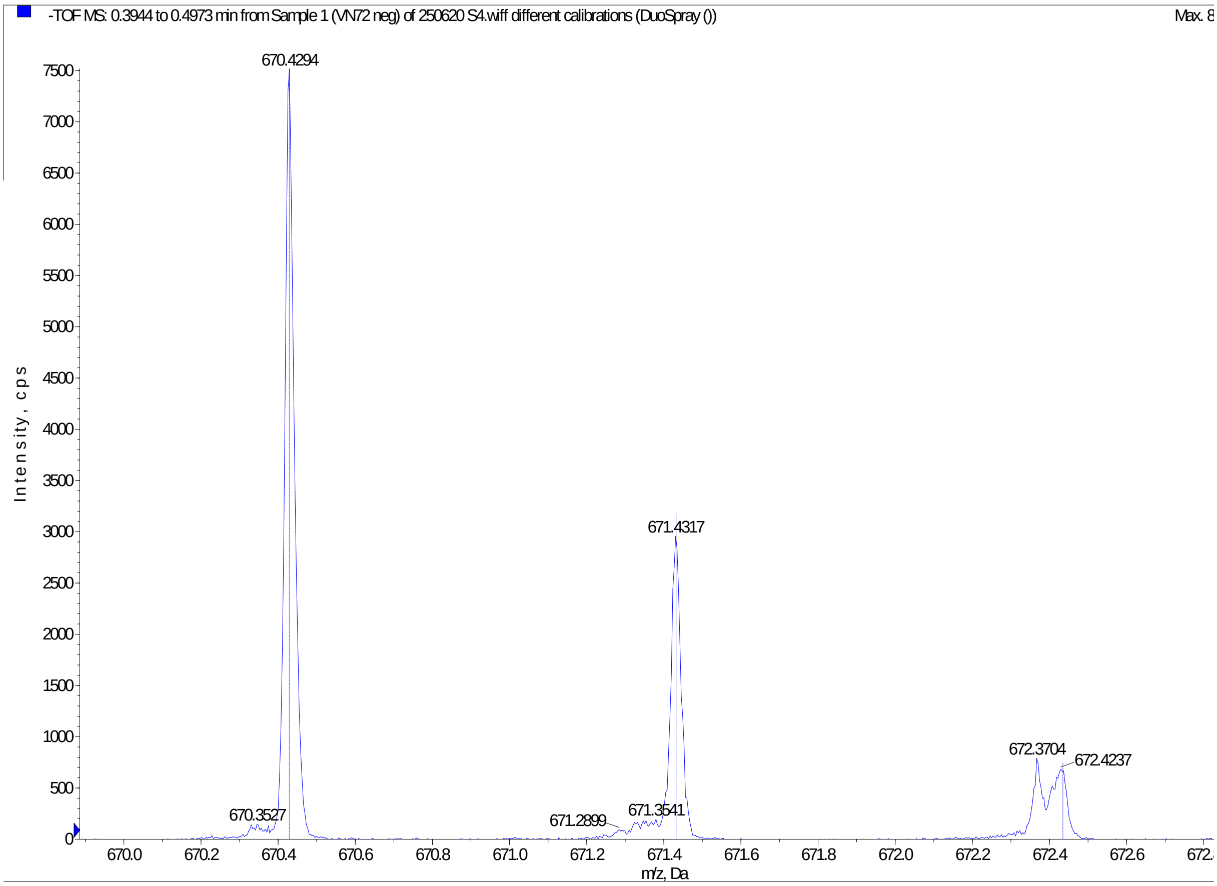

Figure S43. HR-MS spectrum of **2.**

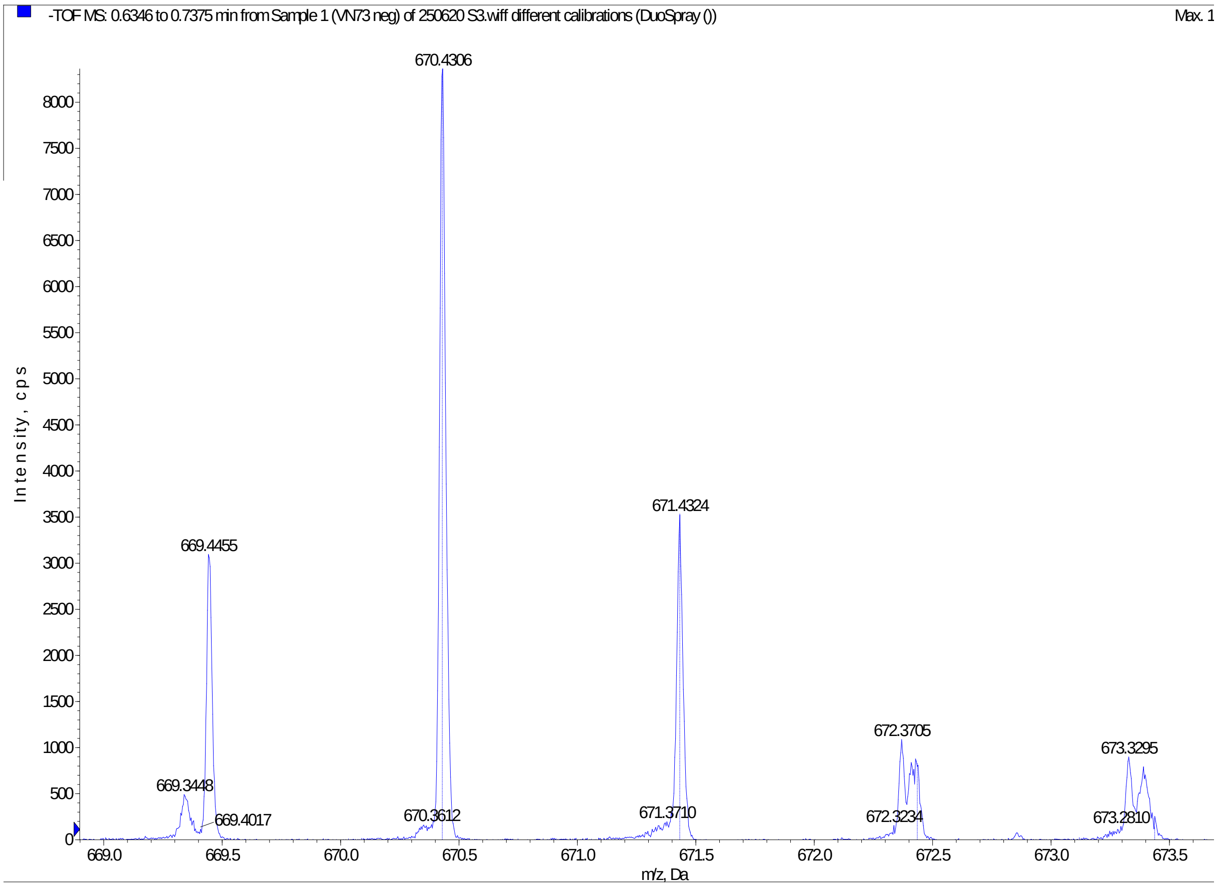

Figure S44. HR-MS spectrum of **3.**

Computational data at the B3LYP/6-311G** level of theory

Cartesian coordinates for **1** E(B3LYP/6-311G**) = -2203.92008782421 a.u.

C	3.88220	-0.93140	4.40420
C	3.48880	-2.32480	4.92610
C	2.15560	-2.05940	5.63400
C	1.41610	-1.12120	4.66430
N	0.74020	-1.85570	3.59510
C	-0.54080	-2.27780	3.71780
C	-1.19510	-2.75330	2.42020
C	-2.03490	-4.04690	2.60160
C	-3.48280	-3.68430	2.19560
C	-3.32660	-2.48800	1.25090
C	-2.21380	-1.67820	1.92270
N	-1.64330	-0.65960	1.05020
C	-1.02570	0.44570	1.52840
C	-0.39260	1.34290	0.47130
C	-0.31330	2.84050	0.85840
C	1.16760	3.26200	0.66620
C	1.76090	2.19090	-0.25930
C	1.08550	0.91170	0.24850
N	1.20640	-0.23300	-0.64440
C	1.56570	-1.47680	-0.23360
C	1.39210	-2.57490	-1.28760
C	2.38580	-3.74320	-1.12030
C	1.59530	-4.99880	-1.51660
C	0.20720	-4.71500	-0.92560
C	-0.04390	-3.22790	-1.22330
N	-0.74600	-3.04440	-2.49580
C	-1.34440	-1.87720	-2.81610
C	-1.83070	-1.71530	-4.24490
C	-3.32990	-1.37590	-4.36070
C	-3.47640	-0.58800	-5.69470
C	-2.03770	-0.22240	-6.14150
C	-1.17970	-0.47320	-4.89520
N	0.25250	-0.61600	-5.18470
O	3.30370	1.71670	5.27680
O	-1.17270	-2.25070	4.76350
O	-0.91280	0.67920	2.72820
O	2.00730	-1.72810	0.88100
O	-1.44570	-0.94660	-2.01510
N	1.40820	1.92680	4.04760
C	2.47440	1.21800	4.53810
C	2.54920	-0.23340	4.04620
C	1.21330	0.03740	-4.50140
O	1.01690	0.75060	-3.53030
C	2.63110	-0.17930	-5.07420
N	3.75800	0.40970	-4.24940
C	3.63760	1.91740	-4.14420
C	3.79740	-0.19460	-2.86090
C	5.05200	0.08050	-4.95520
H	2.03990	-5.91900	-1.13530
H	1.53750	-5.09590	-2.60830
H	0.23820	-4.84810	0.15870
H	-0.59170	-5.35460	-1.30740
H	-3.60280	-0.74780	-3.51030
H	-3.95270	-2.27000	-4.32440
H	-4.08070	0.30730	-5.54430
H	-3.97750	-1.18320	-6.45880

H	-1.94380	0.80270	-6.50330
H	-1.70910	-0.88580	-6.95030
H	4.37300	-0.35340	5.18990
H	4.56270	-0.97000	3.55030
H	4.24700	-2.76030	5.58030
H	3.33090	-3.01960	4.09320
H	2.33340	-1.52820	6.57510
H	1.56500	-2.94950	5.85330
H	1.17310	-1.87540	2.68260
H	-1.98610	-4.39830	3.63160
H	-1.64160	-4.83900	1.95750
H	-4.04320	-3.37220	3.08090
H	-4.02080	-4.52020	1.74420
H	-4.24160	-1.90660	1.11750
H	-2.99720	-2.82410	0.25740
H	-1.71310	-0.77650	0.04610
H	-0.63700	2.98550	1.88840
H	-0.97820	3.42350	0.21810
H	1.68290	3.22660	1.62910
H	1.27040	4.27280	0.26810
H	2.85210	2.12940	-0.21060
H	1.46580	2.36340	-1.30220
H	0.65670	-0.17260	-1.49330
H	2.67740	-3.79870	-0.06940
H	3.29870	-3.60240	-1.70580
H	-0.68410	-3.77350	-3.19020
H	0.50740	-1.17620	-5.98700

H	2.83810	-1.24660	-5.17260
H	2.69340	0.27310	-6.06610
H	4.51280	2.28680	-3.61300
H	3.60980	2.33280	-5.15070
H	2.72860	2.15450	-3.60270
H	4.68600	0.17880	-2.35540
H	2.90580	0.09310	-2.31230
H	3.85570	-1.27820	-2.95580
H	5.87530	0.50420	-4.38350
H	5.16260	-1.00120	-5.01360
H	5.03600	0.51170	-5.95490
H	0.59780	1.45850	3.64860
H	1.24170	2.81620	4.49570
H	2.41260	-0.20970	2.96300
H	0.65070	-0.53850	5.17860
H	-0.42980	-2.89160	1.65230
H	-2.61120	-1.17150	2.80500
H	-0.92140	1.22120	-0.47900
H	1.52880	0.62770	1.20250
H	1.48820	-2.12710	-2.28080
H	-0.65330	-2.75910	-0.45580
H	-1.59140	-2.60030	-4.84720
H	-1.26740	0.35090	-4.18590

Cartesian coordinates for **2** E(B3LYP/6-311G**) = -1997.12887018855 a.u.

N	18.63920	-2.12870	-1.91160
N	16.18970	0.82880	-2.71200
C	17.51470	1.14600	-3.23850
C	18.38430	-0.12120	-3.27240
C	18.47140	-0.78450	-1.89960
O	18.40650	-0.15250	-0.84930
C	17.44980	1.81030	-4.62090
C	19.80340	0.22120	-3.78500
C	19.74690	0.88130	-5.16830
C	18.85200	2.12630	-5.15800
N	15.40990	0.67360	0.84950
C	14.20270	1.22580	0.24170
C	14.22600	1.03520	-1.28520
C	15.52140	1.61440	-1.83900
O	15.92260	2.72370	-1.48930
C	12.91660	0.65490	0.85080
C	11.67960	1.30410	0.21410
C	12.99080	1.70440	-1.92190
C	11.69180	1.15880	-1.31350
N	18.89320	2.05600	0.98790
C	18.60150	1.56020	2.32730
C	17.38020	0.62500	2.28970
C	16.15790	1.35290	1.75150
O	15.84110	2.47400	2.15430
C	19.81760	0.85350	2.94510
C	17.06810	0.08670	3.70410
C	18.28510	-0.61440	4.31940
C	19.50520	0.31400	4.34550
N	17.64040	4.92470	-0.78160
C	19.06830	4.96430	-1.09890
C	19.69230	3.61540	-0.69900
C	19.35670	3.30930	0.76130
O	19.48900	4.15310	1.64120
C	19.35350	5.30260	-2.56330
C	21.21250	3.64560	-0.93860
C	21.52770	3.98840	-2.40130
C	20.86580	5.30550	-2.83250
C	17.08710	5.83340	0.03670
C	15.71610	5.40900	0.59530
N	15.52000	5.85320	2.03720
C	15.34260	7.34890	2.13230
C	16.69520	5.41570	2.89160
C	14.27350	5.18060	2.55130
O	17.60280	6.89570	0.35910
H	18.77100	-2.64240	-2.76780
H	18.83320	-2.59800	-1.03960
H	15.76430	-0.04350	-2.98710
H	17.95470	1.84370	-2.52570
H	16.84850	2.71970	-4.54260
H	16.92700	1.13850	-5.31320
H	17.93320	-0.83260	-3.97890
H	19.36190	0.15830	-5.89770
H	20.75820	1.14320	-5.49060
H	19.31420	2.89420	-4.52910
H	18.77820	2.55030	-6.16240
H	20.41430	-0.68520	-3.81610
H	20.27740	0.89800	-3.06530
H	15.75720	-0.21610	0.52480
H	12.89290	-0.43080	0.69480
H	12.93290	0.82070	1.93120

H	11.66040	2.36990	0.47490	H	19.26110	2.83220	-1.33420
H	14.24110	2.29770	0.43840	H	21.05790	5.49830	-3.89170
H	11.58700	0.09930	-1.57730	H	21.32210	6.13280	-2.27660
H	13.00320	1.54900	-3.00460	H	22.61020	4.04620	-2.54540
H	13.06210	2.78480	-1.75540	H	21.17130	3.17450	-3.04570
H	14.18450	-0.04170	-1.50030	H	21.64770	2.67880	-0.66720
H	18.83160	1.39780	0.21950	H	21.65340	4.39240	-0.27040
H	20.64870	1.56240	2.97540	H	15.17810	7.60420	3.17800
H	20.11730	0.03070	2.28370	H	14.47290	7.63160	1.53990
H	18.35490	2.43220	2.93580	H	16.23690	7.82360	1.74270
H	17.61910	-0.21830	1.63100	H	16.47930	5.70510	3.91910
H	20.37530	-0.21010	4.74980	H	17.59660	5.90290	2.53460
H	19.30530	1.15540	5.02020	H	16.78580	4.33660	2.80620
H	18.04130	-0.95680	5.32900	H	14.09300	5.52350	3.56900
H	18.52240	-1.51050	3.73200	H	14.43030	4.10450	2.53300
H	16.21370	-0.59570	3.65630	H	13.43610	5.46450	1.91490
H	16.76720	0.93100	4.33390	H	15.61240	4.32550	0.58610
H	17.12190	4.07980	-1.01120	H	14.91240	5.85770	0.00890
H	18.91730	6.27710	-2.79730	H	10.76930	0.86530	0.63030
H	18.85330	4.56710	-3.20480	H	10.83360	1.67510	-1.75180
H	19.49630	5.73630	-0.45750				

Cartesian coordinates for the dimer of **3** E(B3LYP/6-311G**) = -4486.79471300043 a.u.

N	6.48370	-0.79830	4.01540
C	6.89320	-2.01610	3.33720
C	8.43610	-2.15690	3.17930
C	8.69720	-2.38070	1.67300
C	7.36450	-2.91760	1.12810
C	6.32720	-2.08870	1.88600
C	4.91870	-2.65580	1.88770
O	4.71390	-3.87180	1.83240
N	3.93270	-1.73840	2.00700
C	2.52680	-2.07390	2.15220
C	1.91010	-1.63130	3.48830
C	0.39880	-1.73500	3.22900
C	0.21260	-1.42820	1.71440
C	1.64110	-1.37400	1.10920
C	1.72430	-2.00200	-0.27510
O	1.85990	-3.21870	-0.44020
N	1.63090	-1.11920	-1.29440
C	1.63110	-1.49000	-2.69860
C	2.30700	-0.41140	-3.59240
C	1.36530	-0.19300	-4.80050
C	0.41930	-1.40410	-4.79160
C	0.20890	-1.67900	-3.29860
C	-0.35500	-3.06000	-3.00280
O	0.06810	-4.06220	-3.58780
N	-1.32040	-3.09100	-2.06460
C	-1.99690	-4.29360	-1.61630
C	-1.76360	-4.65690	-0.14210
C	-2.87990	-5.67140	0.14580
C	-4.09080	-5.20720	-0.71430
C	-3.53230	-4.14960	-1.70600
C	-4.03650	-4.34550	-3.12390
O	-3.75370	-5.35850	-3.77070
N	-4.80330	-3.34470	-3.60930
C	-5.46840	-3.41500	-4.89520
C	-5.60190	-2.05060	-5.58520
C	-6.73500	-2.23910	-6.62050
C	-7.50080	-3.52710	-6.19700
C	-6.93830	-3.91430	-4.82300
C	-7.01600	-5.39920	-4.49730
N	-7.17660	-5.68350	-3.18620
O	-6.91010	-6.27000	-5.36210
C	5.53810	-0.75100	4.97130
C	5.17770	0.66700	5.41600
C	4.31720	0.60370	6.67250
N	4.20400	1.91340	7.44360
C	3.21380	1.70380	8.56160
C	5.53030	2.30080	8.04370
C	3.71000	3.02130	6.55460
O	4.99310	-1.74220	5.46030
H	6.83220	0.07130	3.61360
H	6.49600	-2.84800	3.91770
H	8.94630	-1.26960	3.55820
H	8.78530	-3.00830	3.76760
H	8.92830	-1.42710	1.18960
H	9.53480	-3.05600	1.48640
H	7.26780	-2.81640	0.04450
H	7.23280	-3.97380	1.37980
H	6.33000	-1.06930	1.49330
H	4.16210	-0.75520	2.12410
H	2.43980	-3.15350	2.03000

H	2.25850	-2.23690	4.32690
H	2.20290	-0.59390	3.67850
H	0.05290	-2.74970	3.44550
H	-0.17820	-1.05660	3.86080
H	-0.38790	-2.19580	1.22940
H	-0.30640	-0.48480	1.54430
H	1.95730	-0.33310	1.04040
H	1.42070	-0.14900	-1.06460
H	2.15920	-2.44060	-2.78070
H	2.45370	0.51350	-3.03110
H	3.29530	-0.75390	-3.90520
H	0.78630	0.72370	-4.65700
H	1.90640	-0.09030	-5.74340
H	-0.52020	-1.22310	-5.31910
H	0.89710	-2.27720	-5.24520
H	-0.44130	-0.90640	-2.87870
H	-1.57610	-2.23550	-1.57840
H	-1.67520	-5.11120	-2.26240
H	-0.75840	-5.04480	0.03630
H	-1.89610	-3.75470	0.46060
H	-2.56280	-6.66930	-0.17060
H	-3.12530	-5.73120	1.20820
H	-4.53240	-6.04630	-1.25430
H	-4.87870	-4.76490	-0.10150
H	-3.78590	-3.14710	-1.36030
H	-7.16780	-6.64820	-2.89080
H	-7.22760	-4.95010	-2.48680
H	-5.02310	-2.55790	-3.00210
H	-4.90190	-4.10870	-5.52040
H	-5.88370	-1.30530	-4.83560
H	-4.65840	-1.72640	-6.02900
H	-7.39370	-1.36860	-6.63880
H	-6.32880	-2.35190	-7.62800
H	-8.58200	-3.38070	-6.16160
H	-7.30220	-4.34180	-6.89620
H	-7.43420	-3.35060	-4.03040
H	4.64520	1.14440	4.58910
H	3.14400	2.62170	9.14090
H	2.24670	1.45680	8.12970
H	3.56550	0.88880	9.19050
H	5.38680	3.20010	8.63820
H	5.88080	1.48460	8.67190
H	6.24400	2.49920	7.24950
H	3.53360	3.90410	7.16530
H	4.46460	3.23810	5.80410
H	2.78470	2.69910	6.08270
H	3.29920	0.31010	6.42180
H	4.71480	-0.12610	7.37490
H	6.10240	1.22370	5.57610
N	-9.00090	-1.73030	-1.51520
C	-8.30720	-0.91690	-2.50070
C	-9.22140	-0.11970	-3.43080
C	-8.24970	0.86720	-4.10620
C	-7.10670	1.11810	-3.07520
C	-7.38530	0.17200	-1.88890
C	-6.16180	-0.49160	-1.28640
O	-5.23680	-0.90860	-1.99310
N	-6.19870	-0.67140	0.05040
C	-5.22030	-1.45610	0.77910
C	-5.79250	-2.22500	1.97130
C	-4.52460	-2.67390	2.70750
C	-3.54710	-1.47620	2.58490

C	-4.05410	-0.62370	1.38600	N	-9.39970	-5.70660	1.45170
C	-2.99820	-0.39380	0.32450	C	-9.06400	-7.17020	1.30020
O	-2.36640	-1.34820	-0.13650	C	-8.70040	-5.17580	2.67650
N	-2.81340	0.87650	-0.09110	C	-10.88960	-5.55880	1.61180
C	-1.98490	1.22280	-1.23230	O	-7.42700	-3.36010	-1.43650
C	-2.77540	1.54480	-2.50980	H	-9.82380	-1.34000	-1.07590
C	-1.77510	2.31430	-3.39910	H	-7.68520	-1.59040	-3.08920
C	-0.64420	2.80860	-2.44910	H	-9.74980	-0.76100	-4.13870
C	-1.11570	2.48180	-1.01860	H	-9.96780	0.41660	-2.83420
C	0.02030	2.20120	-0.05340	H	-7.83920	0.41670	-5.01300
O	0.99580	1.51140	-0.37980	H	-8.74970	1.79050	-4.40220
N	-0.08890	2.69460	1.19170	H	-6.13450	0.88460	-3.51080
C	0.92560	2.41930	2.21250	H	-7.07590	2.15500	-2.73780
C	0.52790	3.01570	3.57760	H	-7.95640	0.68950	-1.11290
C	1.29680	4.34120	3.67080	H	-6.96120	-0.26240	0.57110
C	2.65440	3.98670	3.05080	H	-4.79440	-2.16830	0.07350
C	2.29560	3.08570	1.84380	H	-6.39170	-1.55330	2.59720
C	3.37700	2.05850	1.57780	H	-6.42780	-3.05280	1.65330
O	3.63020	1.17310	2.40740	H	-4.71340	-2.95490	3.74490
N	4.05230	2.18810	0.41910	H	-4.10500	-3.54750	2.20000
C	5.18280	1.36450	0.04720	H	-3.55520	-0.86850	3.49140
C	5.20780	0.91640	-1.41400	H	-2.52420	-1.81440	2.42420
C	6.63740	0.37560	-1.57310	H	-4.46080	0.32730	1.73740
C	7.52650	1.26800	-0.65790	H	-3.41950	1.59290	0.28310
C	6.55570	2.07510	0.23320	H	-1.32070	0.37570	-1.40440
C	6.90950	2.11490	1.71040	H	-3.17850	0.64130	-2.96890
O	7.51440	1.18940	2.26240	H	-3.62520	2.17980	-2.23960
N	6.46530	3.18750	2.39320	H	-1.36060	1.66220	-4.17070
C	-8.48060	-2.86950	-1.01970	H	-2.26360	3.14520	-3.91090
C	-9.26550	-3.51550	0.11890	H	0.27930	2.25970	-2.64160
C	-8.91500	-4.99580	0.19800	H	-0.42770	3.87160	-2.56470

H	-1.75300	3.28390	-0.63210	H	8.14930	1.94770	-1.24140
H	-0.87800	3.27870	1.42610	H	6.44660	3.09500	-0.14570
H	1.04500	1.33940	2.27890	H	5.92120	3.91070	1.95030
H	-0.55380	3.11810	3.68960	H	6.56130	3.20890	3.39800
H	0.87110	2.33510	4.36130	H	-10.33430	-3.35810	-0.02780
H	0.79810	5.11280	3.07490	H	-9.37190	-7.69210	2.20330
H	1.38100	4.71280	4.69440	H	-9.59660	-7.56370	0.43750
H	3.24370	4.85460	2.74990	H	-7.99010	-7.26690	1.15680
H	3.24690	3.40690	3.76100	H	-9.03270	-5.75230	3.53680
H	2.14460	3.70830	0.96100	H	-7.62680	-5.28490	2.53900
H	3.77150	2.91850	-0.21890	H	-8.95780	-4.12990	2.81800
H	5.15520	0.48170	0.68340	H	-11.21050	-6.18530	2.44090
H	4.43330	0.17780	-1.62590	H	-11.12480	-4.51920	1.82240
H	5.04090	1.78170	-2.06530	H	-11.37130	-5.87680	0.68940
H	6.67250	-0.66110	-1.22680	H	-9.35140	-5.53250	-0.64380
H	6.97070	0.38240	-2.61190	H	-7.83570	-5.12810	0.16700
H	8.18830	0.66080	-0.04020	H	-8.97760	-2.98190	1.02890
